# Thermal Processing as a Key Factor in the Surface Chemistry and Preliminary Antimicrobial Activity of ZnO Nanoparticles Synthesized Using *Tradescantia spathacea* Sw., Extract

**DOI:** 10.3390/nano16211366

**Published:** 2026-10-26

**Authors:** Jesús Antonio López-García, Eric Jaziel Medrano-Sánchez, Ricardo López-Rodríguez, Ever A. Blé-González, Ammy Joana Gallegos-García, Nelly Cristina Aguilar-Sánchez, María Elena Escalante-Pérez, Felipe Córdova-Lozano, Gerardo Enrique Córdova-Pérez, Alejandro Bugarin, Patricia Álvarez-Fitz, Adrián Cervantes-Uribe, Abraham Gómez-Rivera

**Affiliations:** 1 División Académica de Ciencias Básicas, Universidad Juárez Autónoma de Tabasco, Carretera Cunduacán-Jalpa Km. 0.5, Cunduacán 86690, Mexico; jl122543@gmail.com (J.A.L.-G.); eric.medrano@ujat.mx (E.J.M.-S.); ricardo.lopezr@ujat.mx (R.L.-R.); ever.ble@ujat.mx (E.A.B.-G.); 2 División de Ciencias Básicas e Ingeniería, Universidad Popular de la Chontalpa, Carretera Cárdenas-Huimanguillo Km. 2 S/N, Ranchería, Invitab Paso y Playa, Heroica, Cárdenas 86556, Mexico; joana90102010@gmail.com; 3 División Académica Multidisciplinaria de Jalpa de Méndez, Universidad Juárez Autónoma de Tabasco, Carretera Estatal Libre Villahermosa Comalcalco Km. 27 S/N, Ranchería Rivera Alta, Jalpa de Méndez 86205, Mexico; nelly.aguilar@ujat.mx; 4 División Académica de Ingeniería y Arquitectura, Universidad Juárez Autónoma de Tabasco, Carretera Cunduacán-Jalpa Km. 0.5, Cunduacán 86690, Mexico; maria.escalante.meep@gmail.com; 5 Chemical and Biological Department, Science School, Universidad de las Américas Puebla. Ex hacienda Sta. Catarina Mártir S/N. San Andrés Cholula, Puebla 72810, Mexico; felipe.cordova@udlap.mx; 6 Laboratorio de Innovación y Materiales Aplicados, Facultad de Ciencias Químicas, Benemérita Universidad Autónoma de Puebla, Puebla 72570, Mexico; enrique_cordova90@hotmail.com; 7 Department of Chemistry and Physics, Florida Gulf Coast University, Fort Myers, FL 33965, USA; abugarin@fgcu.edu; 8 Investigadoras e Investigadores por México—Universidad Autónoma de Guerrero, Av. Lázaro Cárdenas s/n. Col. La Haciendita, Chilpancingo 39070, Mexico

**Keywords:** nanoparticles, bio-assisted, *Tradescantia spathacea*, swarming mobility, degradation, 4-nitrophenol

## Abstract

ZnO nanoparticles were synthesized through a bio-assisted route using a hydroalcoholic extract of *Tradescantia spathacea* Sw., a plant previously reported to exhibit antimicrobial activity. Zinc acetate dihydrate [Zn(CH_3_COO)_2_·2H_2_O] was used as the precursor, and the resulting materials were subjected to different thermal treatments (room temperature, 50, 100, 200, and 500 °C) to evaluate changes in their physicochemical and biological properties. The samples were characterized by Fourier-transform infrared spectroscopy (FT-IR), X-ray diffraction (XRD), scanning electron microscopy (SEM), N_2_ adsorption–desorption, UV–Vis diffuse reflectance spectroscopy, and zeta-potential measurements. Their interaction with 4-nitrophenol (4-NP) under dark conditions was also evaluated as a molecular probe of the solid-solution interfacial environment. Antimicrobial activity was assessed against bacterial and fungal microorganisms, while anti-virulence activity was evaluated through the inhibition of swarming motility in *Proteus mirabilis* 884. FT-IR analysis showed that organic species derived from the extract remained in the materials treated between room temperature and 200 °C, whereas these contributions disappeared after treatment at 500 °C. XRD confirmed the formation of the hexagonal wurtzite ZnO structure in all samples, while SEM revealed changes in morphology from agglomerated flake-like structures toward more defined particles. Zeta-potential measurements showed distinct negative electrokinetic behavior among the materials, indicating differences in their interfacial surface environments. In addition, contact with 4-NP modified its protonation equilibrium and produced spectral changes compatible with surface-mediated oxidative transformations under dark conditions. The ZnO materials exhibited antimicrobial activity, with a MIC of 320 μg/mL against *P. mirabilis* 884, and markedly inhibited its swarming motility, reaching more than 96% inhibition for one sample at 24 and 48 h. Overall, the results indicate that the bio-assisted synthesis modifies the surface properties of ZnO and support a surface-mediated mechanism in which changes in the interfacial microenvironment and oxidative processes may contribute to microbial inhibition.

## 1. Introduction

Nanoparticles are of great relevance due to their wide application in various fields such as medicine, computer science, materials, textiles, among others; For example, in computing, they have been used in the manufacture of high-density data storage devices and in the improvement of the energy efficiency of electronic components [1,2], in the same way in the textile sector, nanoparticles have been incorporated into fabrics to confer antibacterial and water-repellent properties [3,4], in addition, in the field of materials, they have been used to improve mechanical resistance, thermal conductivity and durability of compounds [5,6]; in the field of medicine, the use of nanoparticles has great potential; for example, recent studies have demonstrated their antimicrobial activity, such as ZnO nanoparticles obtained through bioassisted synthesis showed great activity against Gram-positive and Gram-negative bacteria [7], this activity was attributed to the generation of reactive oxygen species (ROS) on the surface of the nanoparticles, which caused damage to the cell membrane of microorganisms [8,9]; In addition, these nanoparticles can inhibit the growth of fungi and yeasts, expanding their potential in antifungal applications [10,11].

The use of plant extracts such as *Aloe vera*, *Citrullus colocynthis*, and *Zingiber officinale* for the synthesis of ZnO nanoparticles, have shown not only great efficacy in the formation of nanoparticles, but also antimicrobial and antioxidant properties [7,12,13]. This bioassisted synthesis reduced the use of toxic chemicals and allowed the incorporation of bioactive compounds present in plant extracts, which was able to improve the therapeutic properties of the nanoparticles [14,15].

In addition to their antimicrobial activity, ZnO nanoparticles have been shown to be useful in wound healing, thanks to their ability to promote cell regeneration and reduce inflammation [16,17]. They have also been used in the manufacture of sunscreens due to their ability to absorb UV-A and UV-B radiation, offering more effective protection compared to other compounds such as titanium dioxide [18,19].

Therefore, the use of medicinal plant extracts is of great interest to obtain functionalized nanoparticles, since they combine the properties of the metal with the bioactive compounds of the plant. This is especially relevant for nanoparticles with antimicrobial activity, because bacteria are exhibiting resistance to conventional antibiotics, and today it is a health problem that must be addressed. For example, bacteria such as methicillin-resistant *Staphylococcus aureus* (MRSA) and *Pseudomonas aeruginosa* have developed mechanisms of resistance to multiple drugs, reducing therapeutic options [7,11]. Given this, ZnO nanoparticles obtained through bioassisted synthesis have demonstrated broad antimicrobial activity, even against multidrug-resistant strains, thanks to the synergy between ZnO and the phytochemicals present in the plant extract [9,10].

A promising medicinal plant for this purpose is *Tradescantia spathacea* Sw, commonly known as “purple maguey”, which is native to southern Mexico and Central America, this plant and has been used in traditional Mexican medicine to treat infections, skin wounds and respiratory diseases [20], phytochemical studies have identified in its compound extracts as epigallocatechin, flavonoids and phenolic acids, responsible for its antioxidant, antitumor, and antimicrobial activity [21,22], in particular, ethanolic extracts of *T. spathacea* have been reported to inhibit the growth of multidrug-resistant bacteria, including *Acinetobacter baumannii* and *Enterococcus faecalis* [23], suggesting that extracts of this species can be used in the synthesis of ZnO nanoparticles with antimicrobial applications.

In plant-mediated ZnO synthesis, extract composition and processing conditions can influence nucleation, growth, morphology, and surface-associated organic species, thereby affecting the properties relevant to biological performance [24]. ZnO antimicrobial effects are considered multifactorial and may involve contact with the cell envelope, reactive oxygen species, and Zn^2+^ release; however, the relative contribution of these pathways depends on the material and test conditions and should not be assumed without direct evidence [25].

Although the phytochemical and antimicrobial properties of *T. spathacea* have been reviewed [26], less is known about how post-synthesis thermal treatment affects the surface chemistry and antimicrobial performance of ZnO prepared using this plant. Heating may alter extract-derived organic species and particle organization, while studies of plant-mediated ZnO show that these characteristics depend on the extract and preparation conditions [24,27].

Therefore, this study aimed to investigate the influence of thermal treatment on the physicochemical properties and antimicrobial performance of ZnO nanoparticles synthesized through a bio-assisted route using a hydroalcoholic extract of *T. spathacea*. By evaluating materials obtained at different temperatures, this work seeks to establish relationships between thermal processing, surface-associated organic species, and antimicrobial activity against clinically relevant microorganisms, providing insights into the potential of plant-mediated ZnO nanoparticles as an alternative approach for addressing antibiotic resistance.

## 2. Materials and Methods

### 2.1. Obtaining the Hydroalcoholic Extract

The leaves of *T. spathacea* were collected in the Huapacal 1st section rancheria located in the municipality of Jalpa de Méndez, Tabasco, Mexico, on 25 June 2024, which were washed with distilled water to remove any impurities present, then an infusion was made with a hydroalcoholic system in a ratio of 7:3 (H_2_O: EtOH), using 1.275 Kg of the plant material, this mixture was filtered and proceeded to concentrate in a Rotavapor (BUCHI-R100, Flawil, Switzerland) to eliminate the solvents used to obtain the extract obtaining a total of 5.851 g which was stored protected from humidity and light.

### 2.2. Bio-Assisted Synthesis of ZnO Nanoparticles (NPs)

**Figure 1:**
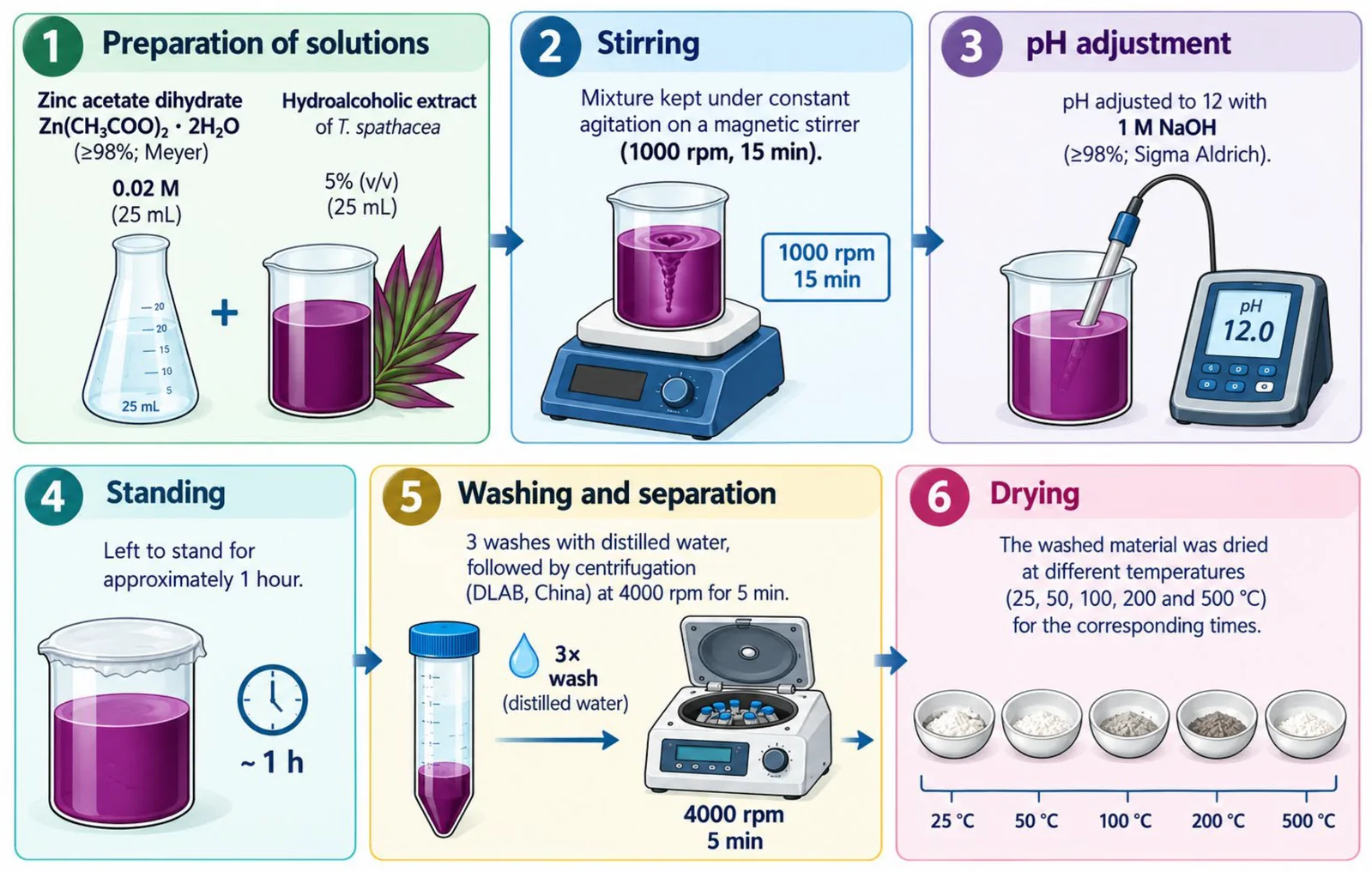
Schematic illustration of the overall synthesis process and key experimental steps for bio-assisted ZnO NPs.

Figure 1 shows the process of bioassisted synthesis of ZnO NPs using the hydroalcoholic extract of *T. spathacea*. ZnO NPs were synthesized from a solution of zinc acetate dihydrate (Zn(CH_3_COO)_2_ • 2H_2_O, (≥98%; Meyer, Mexico)) 0.02 M; 25 mL of a solution of hydroalcoholic extract of *T. spathacea* 5%, then this mixture was kept in constant agitation on a grill with magnetic agitation at 1000 rpm for 15 min, then the pH of the solution was adjusted to 12, with a solution of 1 M NaOH (≥98%; Sigma Aldrich, Mexico), and left to stand for approximately 1 h, then 3 washes were carried out with distilled water and subsequent separation in a centrifuge (DLAB, Beijing, China) at 4000 rpm for 5 min. This process of bioassisted synthesis is led out a total of 5 times in which temperatures and drying time were varied, a ZnO sample was also added which was obtained from zinc nitrate Zn(NO_3_)_2_ which was dissolved in 100 mL of distilled water, using the necessary amount to obtain 1 g of ZnO. The solution was kept in constant agitation for 12 h. Subsequently, recrystallization was carried out by slow evaporation of the solvent at 60 °C for 24 h. The material obtained was calcined at 500 °C, using a heating ramp of 2 °C/min, and this temperature was maintained for 4 h [28].

Table 1 shows the synthesis conditions and the asginated nomenclature for the ZnO NPs obtained from the hydroalcoholic extract of *T. spathacea*.

### 2.3. Characterization Methods

#### 2.3.1. Fourier Transform Infrared Spectroscopy (FT-IR)

The analysis of the functional groups of the ZnO NPsNPs was carried out with the help of a Fourier Transform Infrared Spectroscopy (FTIR) equipment with a PerkinElmer UATR Two™ system equipped with a diamond crystal for Total Attenuated Reflectance (ATR). The measurements covered the spectral range from 4000 to 400 cm^−1^, the samples were deposited in minimal quantities on a Hastelloy plate and uniformly compressed with a metal tip to ensure optimal contact with the crystal.

#### 2.3.2. X-Ray Diffraction (XRD)

X-ray diffraction (XRD) characterization was performed on a Bruker D2 PHASER (Bruker AXS GmbH, Karlsruhe, Germany) diffractometer using a Cu Kα radiation source (*λ* = 0.15418 nm), with a scanning range of 20° to 80° for 660 s. Phase identification was carried out using the JADE 6 database, and the average crystal size was estimated using the Scherrer equation (Equation (1)) [29].

(1) $$D\;=\;\frac{k\lambda }{\mathrm{\beta cos}(\theta )}$$

where *k* is Scherrer’s constant, is the wavelength of the radiation, is the half-maximum width (FWHM), and is the diffraction angle in radians. *λβθ*.

#### 2.3.3. Scanning Electron Microscopy (SEM)

The size and shape of the NPs were observed using a TESCAN MAIA3 high-resolution scanning electron microscope (MAIA3, TESCAN GROUP, a.s., Brno, Czech Republic) equipped with an In-Beam SE detector. The samples were placed on stainless steel supports using carbon tape, and the excess material was removed with a rubber blower. The images were acquired on scales between 20 μm and 5 μm.

#### 2.3.4. N_2_ Adsorption-Desorption

Finally, the textural properties of the materials such as the specific surface area, the pore diameter and the volume of the pore were obtained by nitrogen physisorption with a MICROMERITICS TRISTAR 3020 II equipment (Micromeritics Instrument Corporation, Norcross, GA, USA) at 77 K (−196 °C), the 0.1 g samples were subjected to a degassing process at 300 °C for 3 h. The data obtained were analyzed with the BET method (Brunauer–Emmett–Teller), and the pore size distribution was calculated using Density Functional Theory (DFT).

#### 2.3.5. UV-Vis Diffuse Reflectance Spectroscopy (DRS)

Diffuse reflectance UV–Vis spectra of the ZnO samples were recorded using a Varian Cary 300 spectrophotometer (Varian, Palo Alto, CA, USA) equipped with an integrating sphere. The measurements were carried out over the wavelength range of 200–800 nm using BaSO_4_ as the reflectance reference. The spectra were used to compare the optical response of the bio-assisted ZnO samples (**NTS-1**–**NTS-5**) with that of the chemically synthesized reference (**NC-6**), with particular attention to changes in the reflectance profile associated with the different synthesis and thermal-treatment conditions.

#### 2.3.6. Zeta Potential Measurements

For zeta potential measurements, 10 mg of each sample were dispersed in 10 mL of deionized water in 15 mL Falcon tubes. The suspensions were subjected to ultrasonic treatment at 42 kHz for 380 s to promote particle dispersion. The measurements were performed at pH 6 and 23 °C under identical experimental conditions for all samples. Prior to analysis, the capillary cell was rinsed three times with deionized water. The suspensions were then slowly introduced into the cell (DTS0012 cells in a Zetasizer nanoZS (Malvern Instruments Ltd., Worcestershire, UK) using a 3 mL syringe, taking care to avoid the formation of air bubbles. Three consecutive measurements were performed for each sample, with a total acquisition time of 60 s per measurement. Each measurement consisted of 10 instrumental readings, from which the mean zeta-potential value was calculated.

#### 2.3.7. Interaction with 4-Nitrophenol as a Molecular Probe

The interaction of the ZnO materials with 4-nitrophenol (4-NP) was evaluated using an aqueous 4-NP solution at an initial concentration of 10 mg/L. For each experiment, 200 mL of the solution were contacted with the corresponding ZnO sample at a catalyst concentration of 0.5 g/L. The suspensions were maintained under continuous stirring in complete darkness to exclude photoinduced processes. Preliminary contact-time tests were performed to verify stabilization of the spectral response, and a contact time of 90 min was selected because no appreciable spectral changes were observed thereafter. After the dark-contact period, aliquots were collected and filtered using filter discs with a pore size of 200 μm to remove suspended solid particles. The resulting solutions were analyzed by UV–Vis spectroscopy using a NanoLog^®^ spectrophotometer (HORIBA Jobin Yvon, Longjumeau, France). The spectra obtained before and after contact with the ZnO materials were compared to evaluate changes in the spectral response of 4-NP associated with modifications of the chemical environment at the solid-solution interface.

### 2.4. Antibacterial Activity

#### 2.4.1. Minimum Inhibitory Concentration

The ZnO NPs were evaluate To determine the antimicrobial activity the broth microdilution assay (MIC) described by Gómez Rivera et al. 2026 [30] was used with slight modifications, where the bacterial strains were: (**1**) *Proteus mirabilis* 884, (**2**) *Salmonella dublin* 9676, (**3**) *Escherichia coli* IEC-181-2, (**4**) *Escherichia coli* (clinical isolate), (**5**) *Escherichia coli* GT073, (**6**) *Staphylococcus scuri* 29067 (ATCC), (**7**) *Staphylococcus aureus* UEGE-923, (**8**) *Streptococcus agalactiae* 208310265 and (**9**) *Candida tropicalis* (isolate). The cultures were reseeded on an agar with antibiotic No.1 (Bioxon, Mexico) for 24 h at 37 °C. The strain that was clinically isolated was provided by the General Hospital of Acapulco, State of Guerrero, Mexico, Bacteria Bank of the Autonomous University of Guerrero (UAGro). For the trials, cultures with an incubation of 24 h at 37° C were used, from which 3–4 colonies were taken from each and then diluted in Müller-Hinton broth (MHb; Bioxon, Toluca, Mexico). The inoculums obtained were adjusted using a scale of 0.5 MacFarland (1.5 × 108 CFU/mL). Subsequently, a dilution with distilled water was carried out to obtain 1 × 104 CFU/mL. The minimum inhibitory concentration (MIC) of the nanoparticles was determined by the broth microdilution method [31]. Samples of 50 mg/mL were taken and dissolved in a DMSO-water solution (20:80) and the concentrations evaluated were 320 and 3200 μg/mL. The samples were added to previously sterilized 96-well microplates to which 200 μL of MHb and 2 μL of inoculum (1 × 10^4^ CFU/mL) were incorporated. The controls used were: MHb + DMSO + inoculum and MHb + inoculum; Gentamicin (C+, 100 μg/mL; Sigma Aldrich, Mexico) was used as the reference antibiotic. The microplates were incubated at 37 °C for 24 h, after incubation the MIC was determined by adding 30 μL of a solution (0.05%) of 3-(4,5-dimethylthiazole-2-yl)-2,5-diphenyltetrazolium bromide (MTT, Sigma-Aldrich, Hong Kong, China) in each of the wells, according to the color changes the bacterial viability was determined, being purple if there was viability and colorless if there was not. Each essay was done in a triplicate.

#### 2.4.2. Determination of Antivirulence Activity

The antivirulence activity of the biosynthesized ZnO nanoparticles was evaluated by the swarming mobility assay using the *Proteus mirabilis* 884 strain, according to the methodology presented by Villarreal Araujo et al. (2026) [32] with minor modifications. The nanoparticles (**NTS-1**, **NTS-2**, **NTS-3**, **NTS-4**, **NTS-5** and **NC-6**) were dissolved in 1000 μL of sterile water and aliquots of concentrations 4, 8, 16 and 32 mg/mL were taken and incorporated into Petri dishes with Mueller–Hinton agar (MHb; Bioxon, Toluca, Mexico) supplemented with 1.5% glucose. Once the medium was solidified, 5 μL of a bacterial suspension of *P. mirabilis* 884 (7.5 × 10^5^ CFU) were inoculated in the center of each Petri dish, these were incubated at 37 °C, bacterial motility was evaluated at 24 and 48 h. A digital vernier (Proconsa, Monterrey, Mexico) was used to measure the expansion diameter. As a negative growth control, Mueller–Hinton agar with 1.5% glucose inoculated with the bacterium and without treatment was used, in the same way amikacin (AMK obtained from Pharmaceutical Marketing Company of Chiapas S.A.P.I de C.V. (Mexico city, Mexico)) an antibiotic was used as a positive control at a concentration of 0.37 μg/mL. All determinations were made in triplicate. The percentage of inhibition of swarming mobility was calculated using the following equation (Equation (2)):

(2) $$\%\;\text{Inhibition}=[1-(\frac{\mathrm{Dt}}{\mathrm{Dc}})]\;\times \;100$$

where *Dt* corresponds to the mobility diameter observed in the treatment and *Dc* to the mobility diameter of the negative control. The anti-swarming activity of the nanoparticles was expressed as a percentage of inhibition with respect to the growth control.

## 3. Results

### 3.1. Fourier Transform Infrared Spectroscopy (FT-IR)

**Figure 2:**
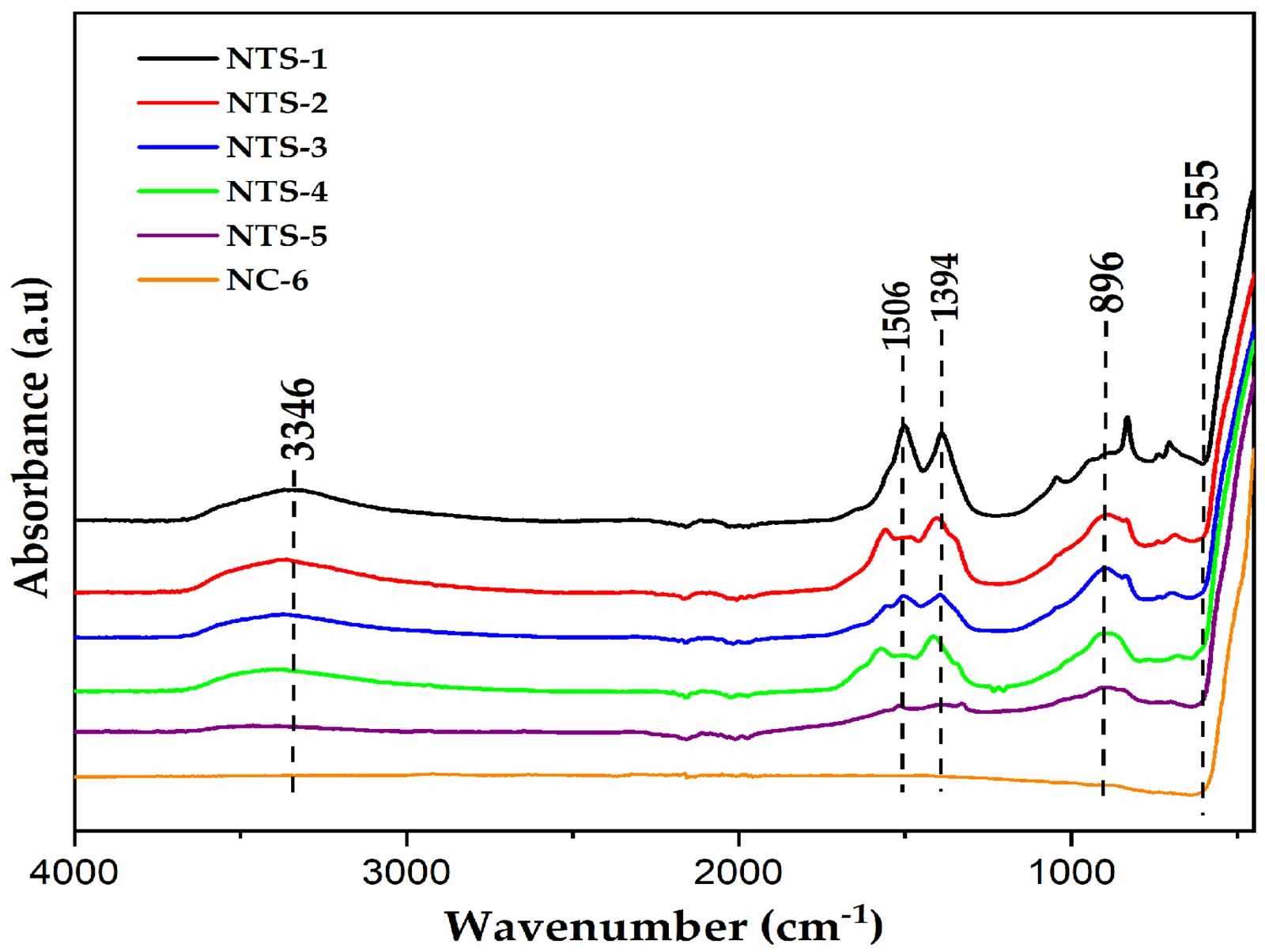
FTIR spectrum of ZnO NPs obtained by bioassisted synthesis using hydroalcoholic extract of *T. spathacea,* where **NTS-1**: room temperature; **NTS-2**: 50 °C; **NTS-3**: 100 °C; **NTS-4**: 200 °C; **NTS-5**: 500 °C; **NC-6**: Combustion at 500 °C.

The use of the hydroalcoholic extract of *T. spathacea* yielded materials whose surface composition and structural characteristics varied depending on the heat treatment conditions. (Figure 2) shows the FT-IR spectra of the ZnO NPs. Differences were observed in the intensity of the signals associated with functional groups among the samples subjected to different temperatures, with **NTS-1**, **NTS-2**, and **NTS-3** exhibiting more prominent signals at 3346 cm^−1^, which can be attributed to the O–H stretching of hydroxyl groups, as well as signals in the 1506–1394 cm^−1^ region corresponding mainly to C–C stretching vibrations in aromatic rings; a prominent peak at 896 cm^−1^ was also observed, which is attributed to C–H bond vibrations [33]. The presence of these signals in the samples subjected to relatively mild thermal conditions suggests the persistence of organic species derived from the bio-assisted synthesis system, together with contributions from adsorbed water [34]. In contrast, the sample treated at higher temperatures (**NTS-4** and **NTS-5**) exhibited a decrease in the intensity of these bands, indicating a progressive reduction in the organic contribution as the severity of the thermal treatment increased [35]. Finally, the absorption band observed below 600 cm^−1^ was attributed to Zn-O vibrations and was detected in all samples.

These results provide evidence for two main features of the synthesized materials: the formation of ZnO and the persistence of organic species associated with the hydroalcoholic extract of *T. spathacea* under relatively mild thermal conditions. However, FT-IR analysis alone does not establish whether these organic species from a coating on the ZnO surface. Therefore, their specific location and interaction with ZnO cannot be conclusively determined from the present data.

### 3.2. X-Ray Diffraction

**Figure 3:**
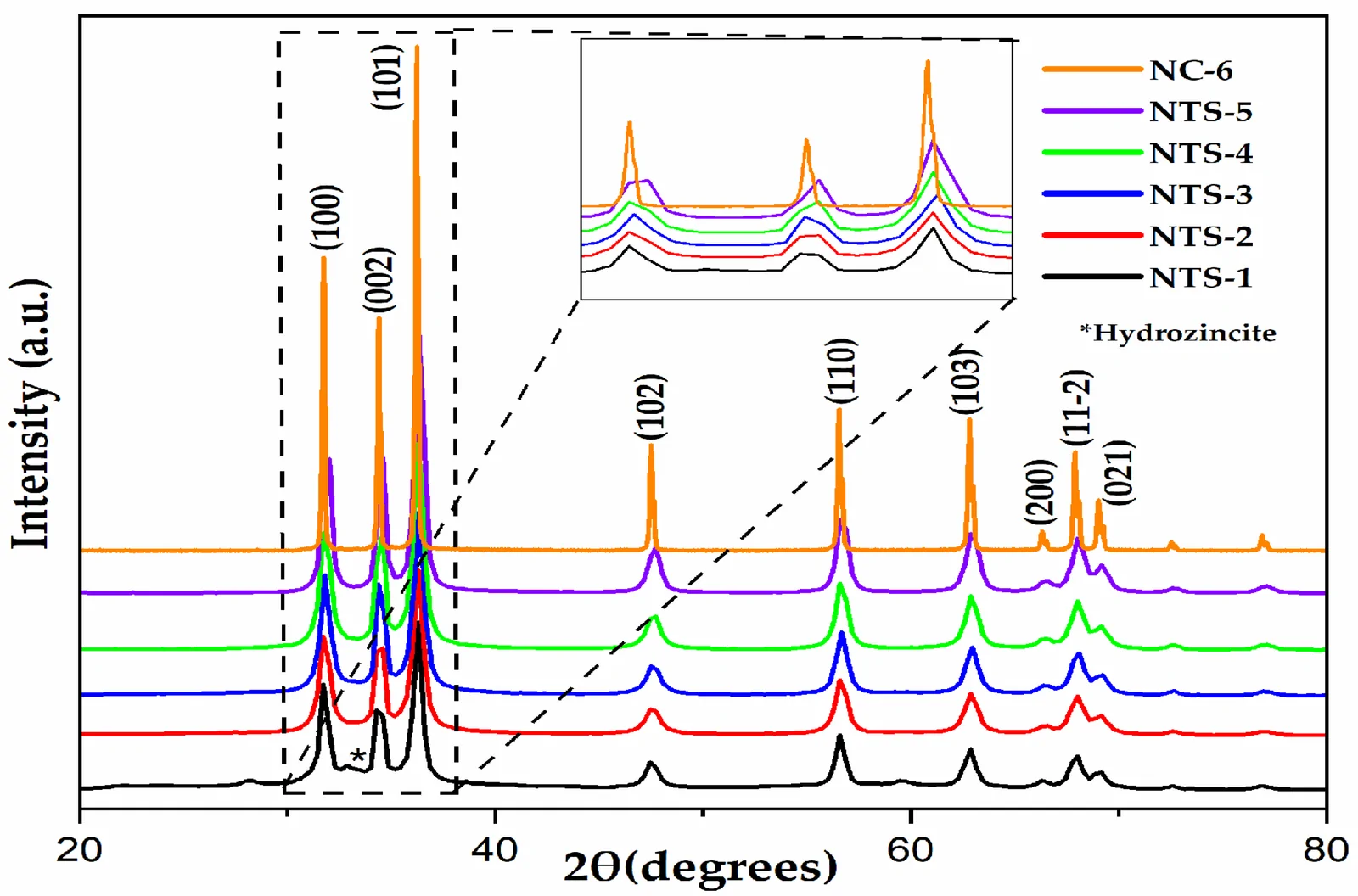
X-ray diffraction patterns of ZnO NPs obtained by bioassisted synthesis using hydroalcoholic extract of *T. spathacea,* where **NTS-1**: room temperature; **NTS-2**: 50 °C; **NTS-3**: 100 °C; **NTS-4**: 200 °C; **NTS-5**: 500 °C; **NC-6**: Combustion at 500 °C.

XRD analyses (Figure 3) showed that ZnO nanoparticles exhibit a wurtzite-like hexagonal crystal structure, given by the characteristic peaks at 31°, 34°, 36°, 47°, 56°, 62°, 66°, 68° and 69° (2θ) corresponding to (100), (002), (101), (102), (110), (103), (200), (11-2) and (021) respectively [36] (JCPDS file no. 36–1451) [7]. In addition, a secondary phase was identified in the **NTS-1** sample belonging to the zinc carbonate hydroxide (hydrozincite) structure, (Zn_5_(CO_3_)_2_(OH)_6_, JCPDS card no. 19–1458); this sample not having heat treatment of any kind led to the formation of this secondary phase [37]. It was also observed that the other samples treated with high temperatures have a high purity of ZnO.

The crystallite size was calculated using the Scherrer equation [29] from the (101) reflection near 36° (2θ), yielding values within a narrow range for the bio-assisted samples, which indicates that the post-synthesis conditions did not substantially modify the average crystallite size. However, small shifts in the position of the main ZnO reflections were observed across the series. To further evaluate these structural variations, the lattice parameters were calculated from the (100), (002), and (101) reflections, and the resulting values are summarized in Table 2.

Although the wurtzite ZnO structure was preserved throughout the series, measurable variations in the lattice parameters were observed. **NTS-5** exhibited the lowest parameter and the smallest unit-cell volume, indicating a contraction of the basal lattice compared with the other materials. In contrast, variations in the c parameter were observed throughout the series, with **NTS-2** and **NTS-4** showing lower values than **NTS-1**, **NTS-3**, and **NC-6**. The lack of a uniform variation in a and c indicates that the structural changes are anisotropic rather than a simple homogeneous expansion or contraction of the ZnO lattice.

These subtle variations in lattice dimensions are consistent with the shifts observed in the diffraction peaks and indicate that the different post-synthesis conditions modify the local crystal environment without changing the predominant wurtzite phase.

### 3.3. Scanning Electron Microscopy (SEM)

**Figure 4:**
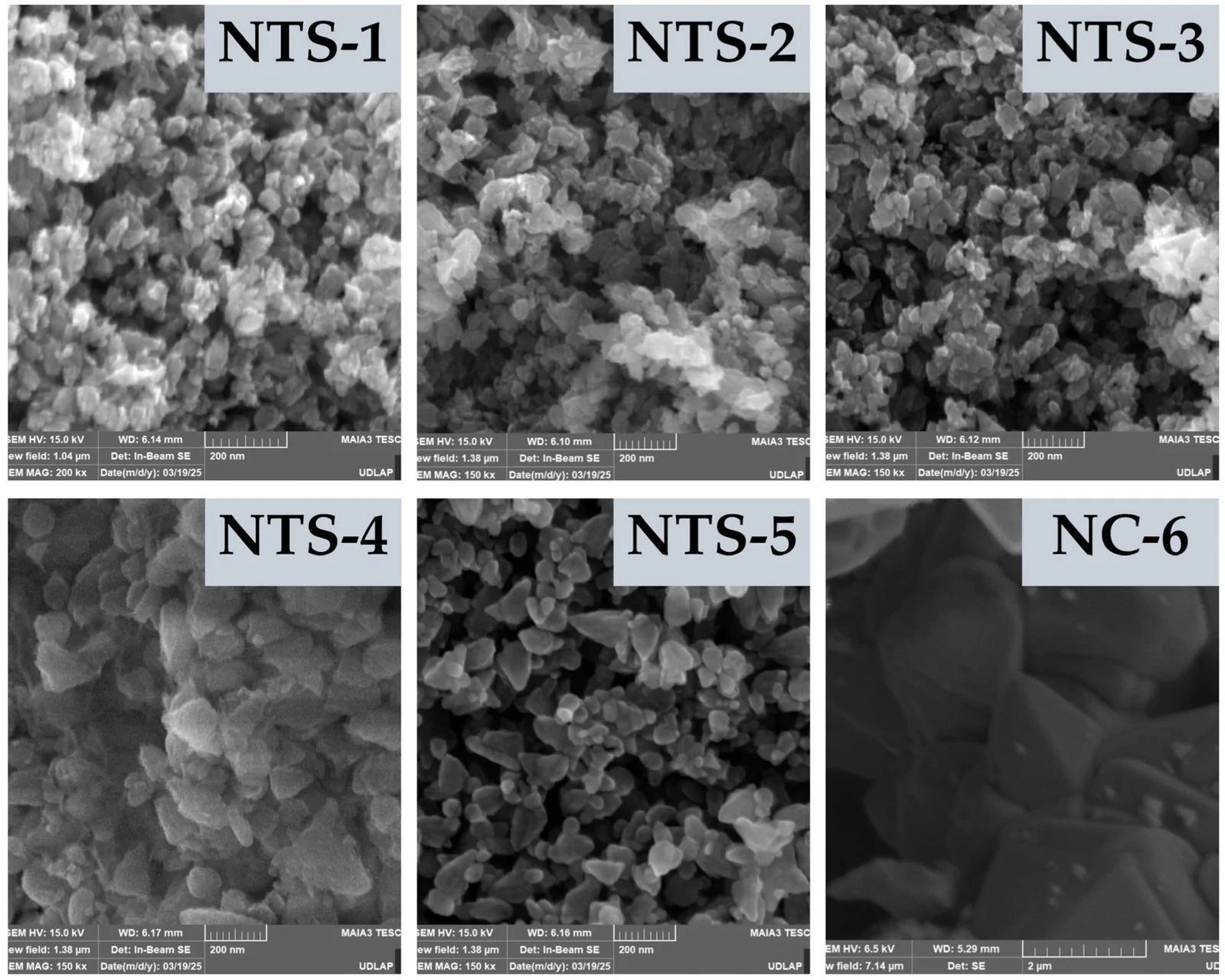
Scanning electron micrographs of ZnO NPs obtained by bioassisted synthesis using hydroalcoholic extract of *T. spathacea,* where **NTS-1**: room temperature; **NTS-2**: 50 °C; **NTS-3**: 100 °C; **NTS-4**: 200 °C; **NTS-5**: 500 °C; **NC-6**: Combustion at 500 °C.

The SEM micrographs (Figure 4) reveal morphological differences between bio-assisted ZnO nanoparticles obtained under different post-synthesis conditions. Samples of **NTS-1**, **NTS-2**, and **NTS-3** exhibit irregularly shaped particles. Agglomeration is particularly evident in **NTS-1** and **NTS-2**, where the particles form compact clusters. A more noticeable morphological distinction is observed in **NTS-4** and **NTS-5**, where the structures resemble triangles and stacked scales, which are easily distinguishable. In **NTS-4**, these structures appear relatively compact and clustered, while **NTS-5** exhibits a more heterogeneous arrangement of particles, suggesting that the bio-assisted synthesis using the hydroalcoholic extract of *T. spathacea* can modulate nanoparticle growth and favor the development of more defined morphologies. This behavior could be attributed to the stabilizing and growth-modulating action of phytochemical compounds present in the extract, which may limit coalescence during nanoparticle formation; however, a quantitative morphological analysis would be required to establish this effect more precisely. Finally, the **NC-6** sample, synthesized by the combustion method, exhibits larger structures with smooth irregular shapes and smaller particles distributed over their surfaces. This morphology differs from that observed in the NTS series, suggesting that both the synthesis route and the post-synthesis treatment conditions influence the final morphology of the ZnO materials.

### 3.4. N_2_ Adsorption–Desorption

**Figure 5:**
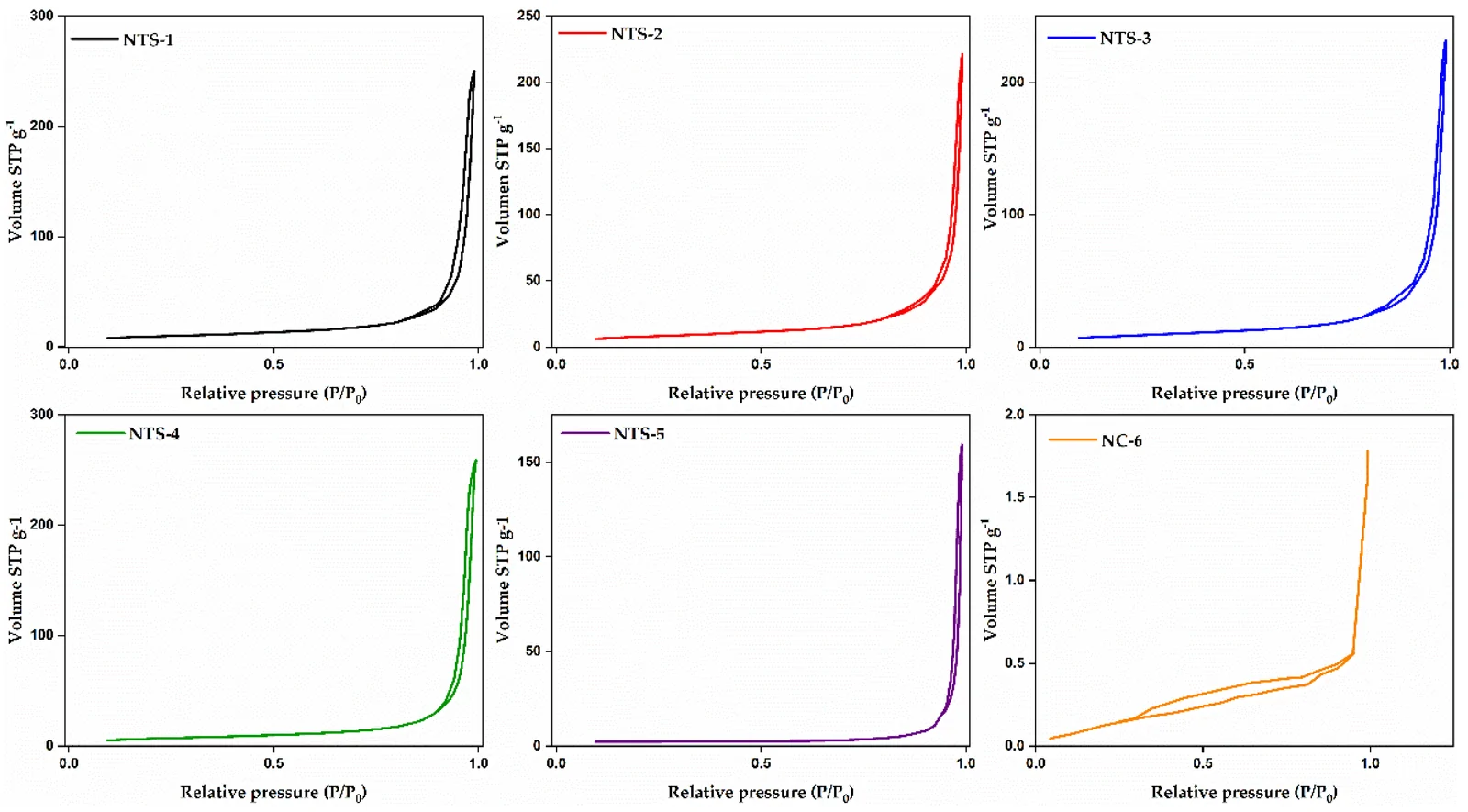
N_2_-absorption–desorption isotherms at 77 K of ZnO NPs obtained by bioassisted synthesis using hydroalcoholic extract of *T. spathacea,* where **NTS-1**: room temperature; **NTS-2**: 50 °C; **NTS-3**: 100 °C; **NTS-4**: 200 °C; **NTS-5**: 500 °C; **NC-6**: Combustion at 500 °.

Nitrogen adsorption–desorption (Figure 5) analysis was used to evaluate the textural characteristics of the ZnO materials. The adsorption–desorption isotherms of the bio-assisted samples exhibited Type IV behavior with hysteresis loops predominantly resembling the H3 type. The relatively low N_2_ uptake at low and intermediate relative pressures, followed by a pronounced increase as P/P_0_ approached unity, indicates that adsorption is mainly governed by multilayer formation followed by capillary condensation within mesoporous and interparticle voids. According to the IUPAC classification, H3-type hysteresis is commonly associated with non-rigid aggregates of particles and pore networks containing large voids that are not completely filled with condensate at saturation [38].

Although the general shape of the isotherms was maintained among **NTS-1**–**NTS-5** differences in the extent of N_2_ adsorption and in the width of hysteresis loops indicate that the post-synthesis thermal-treatment conditions modified the textural organization of the materials. These changes are reflected in the parameters summarized in Table 2. **NTS-1**–**NTS-4** retained relatively similar specific surface areas, whereas **NTS-5** exhibit a marked reduction in accessible surface area together with an increase in average pore diameter. This behavior suggests that the more severe post-synthesis treatment conditions were associated with structural rearrangement and the development of larger interparticle voids. Because both treatment temperature and duration varied among the bio-assisted samples, these changes are attributed to the overall thermal-treatment conditions rather than to temperature alone. **NC-6** exhibited a markedly different adsorption–desorption profile, characterized by type II-like isotherm with an H3-type hysteresis loop. Type II isotherms are generally associated with nonporous or macroporous materials in which unrestricted monolayer-multilayer adsorption occurs, whereas H3 hysteresis can arise from particle aggregates and incompletely filled macroporus or interparticle spaces. The continuous increase in N_2_ uptake near P/P_0_ = 1, without the development of a well-defined saturation plateau, therefore suggests that the adsorption behavior is mainly associated with large interparticle voids rather than with a well-developed mesoporous network.

The textural parameters calculated for **NC-6** are included in Table 2 for completeness; however, the pore volume and average pore diameter should be interpreted with caution. According to the IUPAC recommendations, when an isotherm does not become nearly horizontal at high relative pressure, as occurs in materials containing a significant contribution from macropores, the total pore volume cannot be reliably determined from the amount adsorbed near saturation. Therefore, the values reported for **NC-6** provide an approximate textural estimate rather than a rigorous determination of its total volume and pore diameter.

### 3.5. UV–Vis Diffuse Reflectance Spectroscopy (DRS)

**Figure 6:**
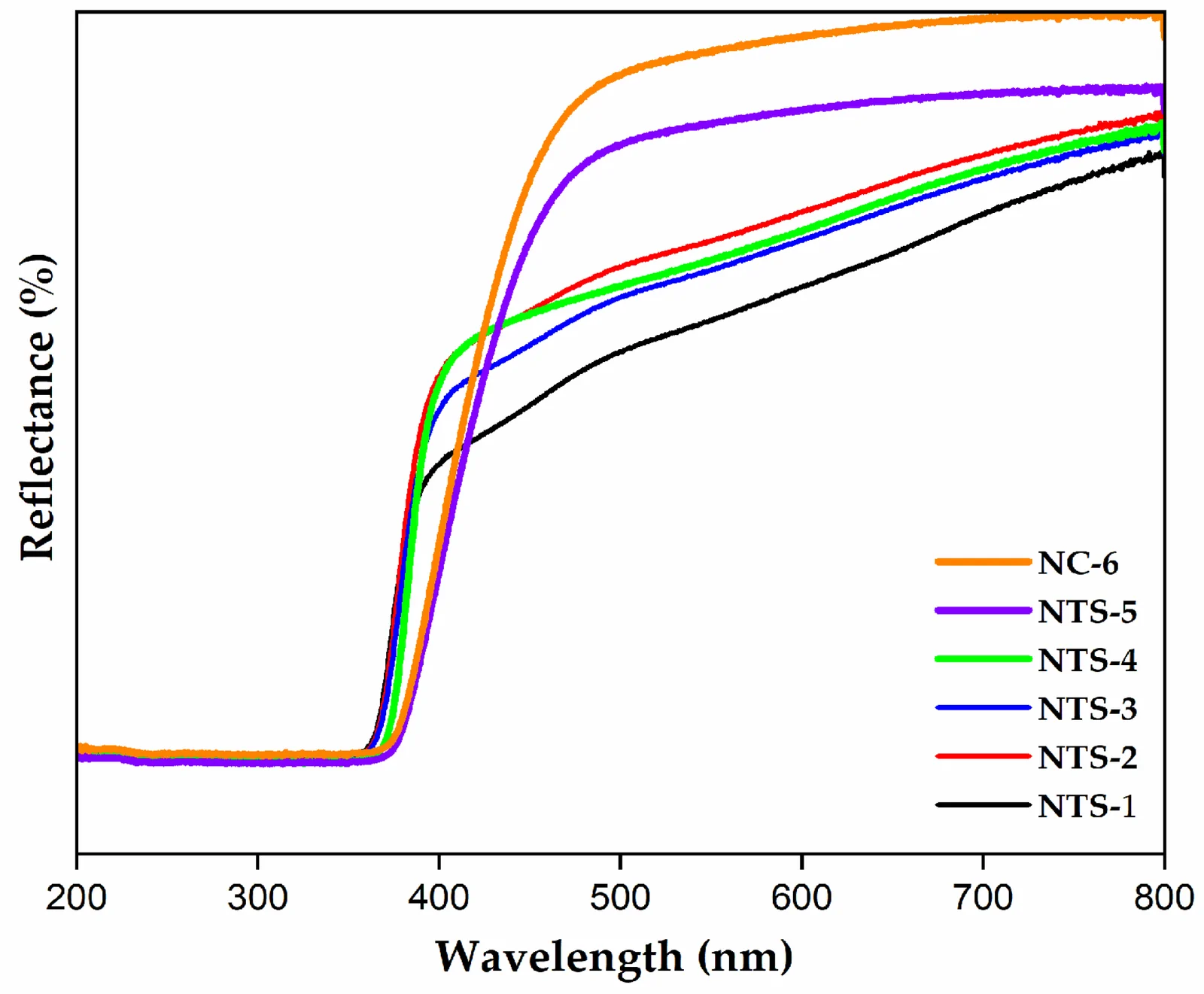
UV–Vis diffuse reflectance spectra of ZnO NPs obtained by bioassisted synthesis using hydroalcoholic extract of *T. spathacea,* where **NTS-1**: room temperature; **NTS-2**: 50 °C; **NTS-3**: 100 °C; **NTS-4**: 200 °C; **NTS-5**: 500 °C; **NC-6**: Combustion at 500 °C.

The UV–Vis diffuse reflectance spectra (Figure 6) show that all samples exhibit a pronounced transition in the near-ultraviolet region, characteristic of ZnO materials. Although the overall position of this transition is preserved throughout the series, marked differences are observed in the reflectance profiles at wavelengths above approximately 400 nm. **NC-6** exhibits the highest reflectance values in the visible region, followed by **NTS-5**, whereas **NTS-1** shows the lowest response. **NTS-2**, **NTS-3**, and **NTS-4** display intermediate and relatively similar behaviors.

These differences indicate that, although the samples retain the characteristic optical response of ZnO, the interaction of the incident radiation with the solids is not equivalent. In particular, the lower reflectance observed for **NTS-1**–**NTS-4** coincides with the samples in which FT-IR analysis evidenced the persistence of organic species associated with the bio-assisted synthesis. In contrast, **NTS-5** exhibits a profile closer to that of **NC-6**, in agreement with the decrease in these organic contributions observed by FT-IR. This behavior suggests that surface modifications associated with the bio-assisted route contribute to the optical differences observed among the samples.

Nevertheless, because diffuse reflectance can also be influenced by light-scattering phenomena, morphology, particle size, and aggregation, these variations cannot be attributed exclusively to surface species. Overall, the results reveal differences in the optical response of the samples that complement the surface and interfacial variations observed by FT-IR and zeta-potential measurements.

**Figure 7:**
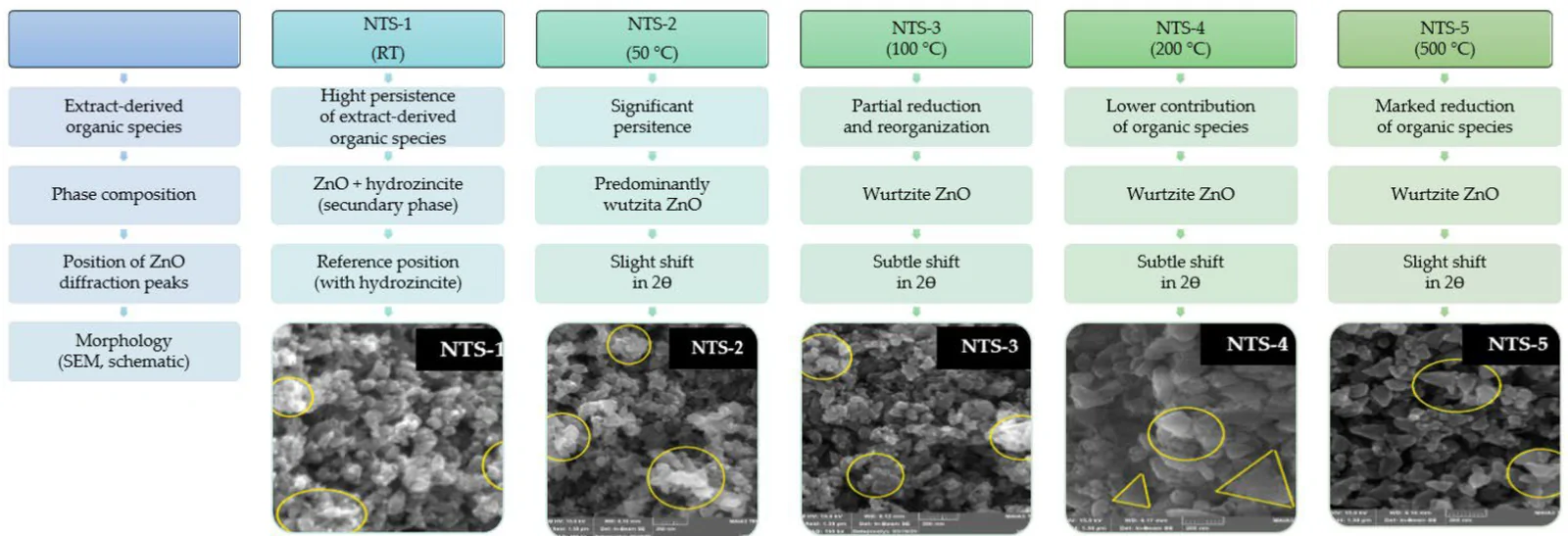
Morphological and structural evolution diagram.

Summarizes the structural and morphological evolution (Figure 7) observed across the NTS series under different post-synthesis conditions. Under the mildest conditions, the materials retain a higher contribution of extract-derived organic species and, in the case of **NTS-1**, a secondary hydrozincite phase is also present. As the post-synthesis treatment becomes more severe, the wurtzite ZnO phase becomes predominant, while subtle shifts in the diffraction peaks indicate small changes in the local crystal structure. At the same time, morphology evolves from irregular and agglomerated particles toward more defined triangular and stacked structures, particularly for **NTS-4** and **NTS-5**. Overall, the diagram illustrates that the post-synthesis conditions affect phase composition, local structural arrangement, and particle morphology simultaneously, rather than producing a single isolated change.

### 3.6. Zeta Potential Measurements

The zeta-potential values indicate that all samples exhibit a negative electrokinetic character under the measurement conditions employed, see Table 2. However, the magnitude of this potential varies among the materials, evidencing differences in their surface environment. **NTS-2** shows the most negative value, followed by **NTS-3** and **NTS-4**, whereas **NTS-1**, **NTS-5**, and **NC-6** exhibit values closer to zero.

Because all measurements were performed under the same experimental conditions, these variations can be associated with differences in the surface chemistry of the materials. In the bio-assisted samples, the presence of extract-derived surface species, together with differences in the protonation state of ZnO surface groups, may contribute to the observed electrokinetic behavior. Nevertheless, zeta potential does not allow the direct identification of the species responsible for the surface charge; therefore, these results should be interpreted as evidence of differences in the interfacial environment rather than as the specific identification of surface functional groups.

The comparison with **NC-6** is particularly relevant. Although this material also exhibits a negative zeta potential, its value is closer to zero than those observed for **NTS-2**, **NTS-3**, and **NTS-4**. This suggests that the bio-assisted synthesis route modifies the surface behavior of ZnO, although the effect is not uniform among the samples.

On the other hand, the absolute magnitudes of the zeta-potential values are relatively low and therefore do not indicate strong electrostatic stabilization of the dispersions. Consequently, these values should not be interpreted primarily in terms of colloidal stability, but rather as a comparative tool for evaluating differences in the surface electrokinetic behavior of the materials.

Overall, the zeta-potential results demonstrate that the materials exhibit distinct surface electrokinetic behaviors under equivalent experimental conditions. These differences indicate that the solid-solution interface is not equivalent among the samples and provide a basis for subsequently analyzing how such surface variations may influence the interaction of the materials with species present in the aqueous medium.

### 3.7. Interaction with 4-Nitrophenol as a Molecular Probe

**Figure 8:**
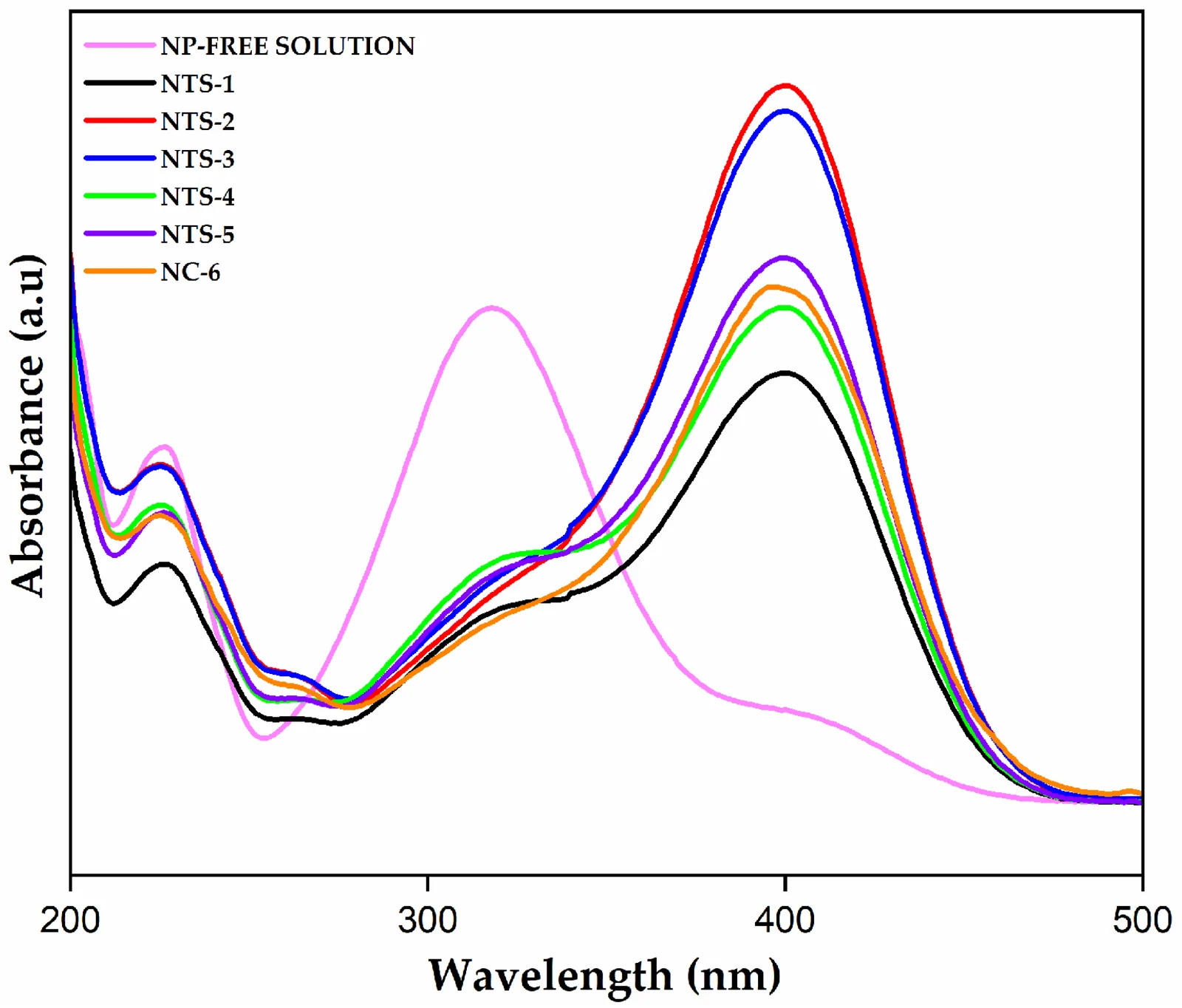
UV–Vis spectra of ZnO NPs obtained by bio-assisted synthesis using hydroalcoholic extract of *T. spathacea* during the interaction with 4-nitrophenol as molecular probe under dark conditions, where **NTS-1**: room temperature; **NTS-2**: 50 °C; **NTS-3**: 100 °C; **NTS-4**: 200 °C; **NTS-5**: 500 °C; and **NC-6**: combustion at 500 °C.

The UV–Vis spectra (Figure 8) obtained after 90 min of contact in the dark show a modification of the spectral response of 4-nitrophenol in the presence of the ZnO materials. The initial solution exhibits absorption bands associated with both the protonated 4-NPOH species and the deprotonated 4-NP^−^ form, in agreement with the acid–base equilibrium of 4-NP under near-neutral pH conditions. Alejandro-López et al. reported that 4-NP has a pKa close to 7.2; therefore, both species may coexist in aqueous solution depending on the pH of the medium [39].

After contact with the **NTS** samples and **NC-6**, the contribution near 400 nm becomes more pronounced, indicating a shift in the spectral response toward the deprotonated 4-NP^−^ species. This behavior suggests that interaction with the ZnO surface modifies the chemical environment experienced by the molecule. A similar effect has previously been described for ZnO, where surface interactions may promote 4-NPOH deprotonation and increase the contribution of 4-NP^−^ in solution [39]. The differences observed among **NTS-1**–**NTS-5** and **NC-6** therefore indicate that, although ZnO itself may participate in this acid–base shift, the magnitude of the effect depends on the surface characteristics of each material. This interpretation is consistent with the zeta-potential values, which reveal distinct electrokinetic behaviors under equivalent experimental conditions.

In addition to the changes in the relative contributions of 4-NPOH and 4-NP^−^, the spectra show modifications in the ultraviolet region around 280–290 nm. This region is relevant because hydroquinone has been identified as one of the main intermediates in 4-nitrophenol transformation pathways. Aman et al. reported the formation of hydroquinone and benzoquinone during 4-NP degradation and confirmed both products by HPLC using detection at 280 nm [40]. Therefore, the appearance of an additional spectral contribution in this region in the systems studied here is consistent with the formation of hydroxylated aromatic species, including hydroquinone.

Taken together, these results indicate that the ZnO surface not only modifies the acid–base equilibrium of 4-NP but may also promote additional interfacial transformations even in the absence of irradiation. The possible formation of hydroquinone is consistent with oxidative pathways reported for 4-NP involving reactive oxygen species. However, since the specific oxidizing species were not directly identified in this study, this assignment is proposed as a mechanistic interpretation supported by the spectral response and previous literature, rather than as an unequivocal identification of a particular ROS.

### 3.8. Antimicrobial Activity

#### Minimum Inhibitory Concentration (MIC)

The ZnO materials inhibited the growth of the microorganisms evaluated, with reported MIC values of 3200 μg/mL for most strains and 320 μg/mL for *Proteus mirabilis* 884 (Table 3). These results indicate a higher susceptibility of *P. mirabilis* 884 under the conditions tested. However, the similar MIC values obtained for the remaining microorganisms do not establish a common mechanism of action. In addition, because extract-only and precursor-related controls were not included, the contribution of plant-derived components to the observed antimicrobial response cannot be determined independently.

From the results obtained by microdilution in broth, it was observed that *P. mirabilis* 884 was the bacterium that presented the highest sensitivity to ZnO nanoparticles, for all samples. These results show that the nanoparticles were able to inhibit the growth of the bacteria evaluated, although the results varied according to the strain because the ability of the bacteria to move may also be related to their infection capacity, so it was considered important to evaluate the effect of the nanoparticles on swarming-type mobility. In this way, it was sought to determine if, in addition to affecting bacterial growth, the nanoparticles could decrease the movement capacity of the bacteria.

**Table 3:** Antibacterial activity (MIC µg/mL) of ZnO NPs.

Experiment				Bacteria					
	**Gram (−)**					**Gram (+)**			**Fungi**
	**1**	**2**	**3**	**4**	**5**	**6**	**7**	**8**	**9**
	µg/mL	µg/mL	µg/mL	µg/mL	µg/mL	µg/mL	µg/mL	µg/mL	µg/mL
**NTS-1**	320	3200	3200	3200	3200	3200	3200	3200	3200
**NTS-2**	320	3200	3200	3200	3200	3200	3200	3200	3200
**NTS-3**	320	3200	3200	3200	3200	3200	3200	3200	3200
**NTS-4**	320	3200	3200	3200	3200	3200	3200	3200	3200
**NTS-5**	320	3200	3200	3200	3200	3200	3200	3200	3200
**NC-6**	320	3200	3200	3200	3200	3200	3200	3200	3200

The labels correspond to the bacteria (**1**) *Proteus mirabilis* 884, (**2**) *Salmonella dublin* 9676, (**3**) *Escherichia coli* IEC-181-2, (**4**) *Escherichia coli* (clinical isolate), (**5**) *Escherichia coli* GT073, (**6**) *Staphylococcus scuri* 29067 (ATCC), (**7**) *Staphylococcus aureus* UEGE-923, (**8**) *Streptococcus agalactiae* 208310265 and (**9**) *Candida tropicalis* (isolated).

### 3.9. Antivirulence Activity

#### Effect of Amikacin and ZnO NPs on Swarming Mobility

Finally, both amikacin (AMK) and the ZnO nanoparticles reduced the swarming mobility of *P. mirabilis* 884 at the concentrations evaluated (Table 4). However, the magnitude of inhibition varied according to the thermal conditions used during the bio-assisted synthesis. At concentrations of 3.2, 1.6, and 0.8 mg/mL, **NTS-2**, **NTS-3**, **NTS-4**, and **NTS-5** produced complete inhibition of swarming under the conditions evaluated. The most relevant differences among the materials were observed at the lowest concentration tested (0.4 mg/mL), where **NTS-4** produced the highest inhibition, reaching 96.69% and 97.58% after 24 and 48 h, respectively (Figures S1 and S2).

In contrast, **NTS-1** showed the lowest inhibitory response, indicating that the absence of thermal treatment resulted in a different biological performance.

These results indicate that the thermal treatment used during the post-synthesis process modulated the anti-swarming activity of the ZnO nanoparticles, although the response was not linearly related to temperature. The highest activity observed for **NTS-4** suggests that the treatment at 200 °C may provide a favorable balance between ZnO formation and the retention of surface-associated organic functionalities derived from the *T. spathacea* extract. In contrast, treatment at 500 °C was associated with a marked reduction in the FT-IR signals attributed to organic components, which may indicate their thermal degradation or removal from the nanoparticle surface. Therefore, the biological response may reflect the combined contribution of the ZnO phase and surface-associated plant-derived components rather than temperature alone.

At 24 h, **NTS-1** showed the ability to reduce bacterial mobility; however, the heat-treated materials generally exhibited greater inhibition. At concentrations of 0.8 mg/mL and above, **NTS-2**, **NTS-3**, **NTS-4**, and **NTS-5** produced complete inhibition under the experimental conditions, and this response was maintained at 48 h. Because these concentrations produced a maximum response in several samples, the lowest concentration tested (0.4 mg/mL) provided a more useful condition for differentiating the biological performance of the nanoparticles.

At 0.4 mg/mL, **NTS-4** showed the highest anti-swarming activity, with 96.69% inhibition at 24 h and 97.58% at 48 h. **NTS-3** also exhibited substantial inhibition, whereas **NTS-5** showed a lower response than **NTS-4**. These findings indicate that the effect of thermal treatment was not simply proportional to temperature. Instead, an intermediate treatment condition, represented by **NTS-4**, was associated with the highest anti-swarming response among the biosynthesized materials.

## 4. Discussion

The differences in anti-swarming activity were considered in relation to the physicochemical characteristics of the ZnO materials. Although the crystallite size remained within a narrow range among the bio-assisted samples, other properties varied considerably, including the persistence of extract-derived organic species, crystalline phase composition, morphology, aggregation, textural characteristics, optical response, and electrokinetic surface behavior. Therefore, the anti-swarming response cannot be attributed to crystallite size or to any single physicochemical parameter.

FT-IR analysis confirmed Zn–O vibrations below 600 cm^−1^ in all samples and revealed that materials obtained under milder post-synthesis conditions retained bands associated with organic species derived from the *T. spathacea* extract. These contributions decreased progressively and were markedly reduced in **NTS-5**. Although FT-IR does not establish the specific location of these organic species or demonstrate the formation of a surface coating, their persistence indicates that the bio-assisted synthesis produced materials with different chemical environments. This interpretation is further supported by the diffuse-reflectance and zeta-potential measurements, which revealed differences in optical response and electrokinetic behavior among the samples.

XRD confirmed the formation of the hexagonal wurtzite-like ZnO phase throughout the NTS series, whereas **NTS-1** additionally exhibited a secondary hydrozincite phase. The crystallite sizes of the bio-assisted samples remained nearly constant, indicating that the differences in biological response cannot be primarily explained by crystallite size. Instead, changes in phase composition and in the chemical characteristics associated with the materials are more likely to contribute to the distinct biological behavior.

SEM analysis also revealed substantial differences in particle organization. **NTS-1**, **NTS-2**, and **NTS-3** predominantly exhibited irregular and agglomerated structures, whereas **NTS-4** and **NTS-5** showed more defined triangular and stacked features. **NC-6**, obtained through the combustion route, displayed a clearly different morphology characterized by larger irregular structures with smaller particles distributed over their surfaces. These observations confirm that the synthesis route and post-synthesis treatment conditions influenced particle morphology and aggregation.

The N_2_ adsorption–desorption results further demonstrated that these morphological differences were accompanied by changes in textural organization. The bio-assisted samples exhibited Type IV isotherms with predominantly H3-type hysteresis, whereas **NC-6** showed a Type II-like profile with an H3-type hysteresis loop. Among the NTS samples, variations were observed in specific surface area, pore volume, and apparent pore size, while crystallite size remained essentially unchanged. Importantly, no direct relationship was observed between a single textural parameter and the anti-swarming response. For example, **NTS-4** exhibited the highest inhibition at the lowest concentration tested without presenting the highest specific surface area. This observation further supports the interpretation that microbial response arises from the combined contribution of several physicochemical characteristics rather than from surface area alone.

The MIC assays showed susceptibility in most of the microorganisms evaluated. *P. mirabilis* 884 was the most susceptible microorganism, with a MIC of 320 μg/mL, while the other bacterial strains and *Candida tropicalis* had values of 3200 μg/mL. These results are consistent with previous findings which described the antibacterial activity of ZnO nanoparticles against different bacterial species [41]. Likewise, a previous study on the plant *T. spathacea* has documented antimicrobial activity of its plant extracts, with variable MIC values depending on the type of extract and the microorganism evaluated [21]. The increased susceptibility of *P. mirabilis* 884 justified the evaluation of its swarm phenotype, which allowed examining the responses to ZnO materials beyond the inhibition of microbial growth. An important limitation of the present study is the absence of extract-only and zinc precursor/NaOH controls. Consequently, the individual contributions of the ZnO phase, extract-derived components, and potential synthesis-related residues to the observed biological responses cannot be separated. Although **NC-6** provides a chemically synthesized ZnO reference, it does not resolve these contributions. Future experiments incorporating these controls are needed to clarify the role of each component.

These findings should also be interpreted in light of reports that ZnO antimicrobial activity varies with the bacterial target and assay conditions, and that several proposed mechanisms may coexist rather than represent a universal pathway [25]. Reviews of plant-mediated metal and metal-oxide nanoparticles further emphasize that extract chemistry and processing conditions influence material properties and complicate direct comparisons across studies [27].

The stronger response of **NTS-4** is consistent with the broader principle that post-synthesis conditions can change the surface-associated chemistry and morphology of plant-mediated ZnO, which may in turn affect contact with microbial cells [24,25]. However, the present experiments do not isolate the contribution of these factors to swarming inhibition. The result is therefore best described as an association between the intermediate treatment condition and the strongest anti-swarming response, rather than proof that a particular surface group or antimicrobial pathway caused the effect.

At the lowest concentration evaluated in the swarming assay (0.4 mg/mL), clear differences among the materials became evident. **NTS-4** exhibited the strongest response, reaching 96.69% and 97.58% inhibition after 24 and 48 h, respectively, whereas the other samples showed lower inhibition under the same conditions. Because both the treatment temperature and duration varied across the NTS series, this behavior should not be attributed exclusively to temperature. Rather, the results indicate that the overall post-synthesis conditions generated materials with different combinations of chemical, morphological, textural, optical, and interfacial properties.

Taken together, these observations show that the physicochemical characteristics of the materials vary across the series and are associated with differences in the observed swarming response. However, the present data does not establish a direct causal relationship between any individual material property and the biological outcome. These findings provide a basis for proposing, but do not experimentally confirm, possible surface-mediated interactions that may contribute to the observed effects.

### Proposed Surface-Mediated Mechanism of Microbial Inhibition

**Figure 9:**
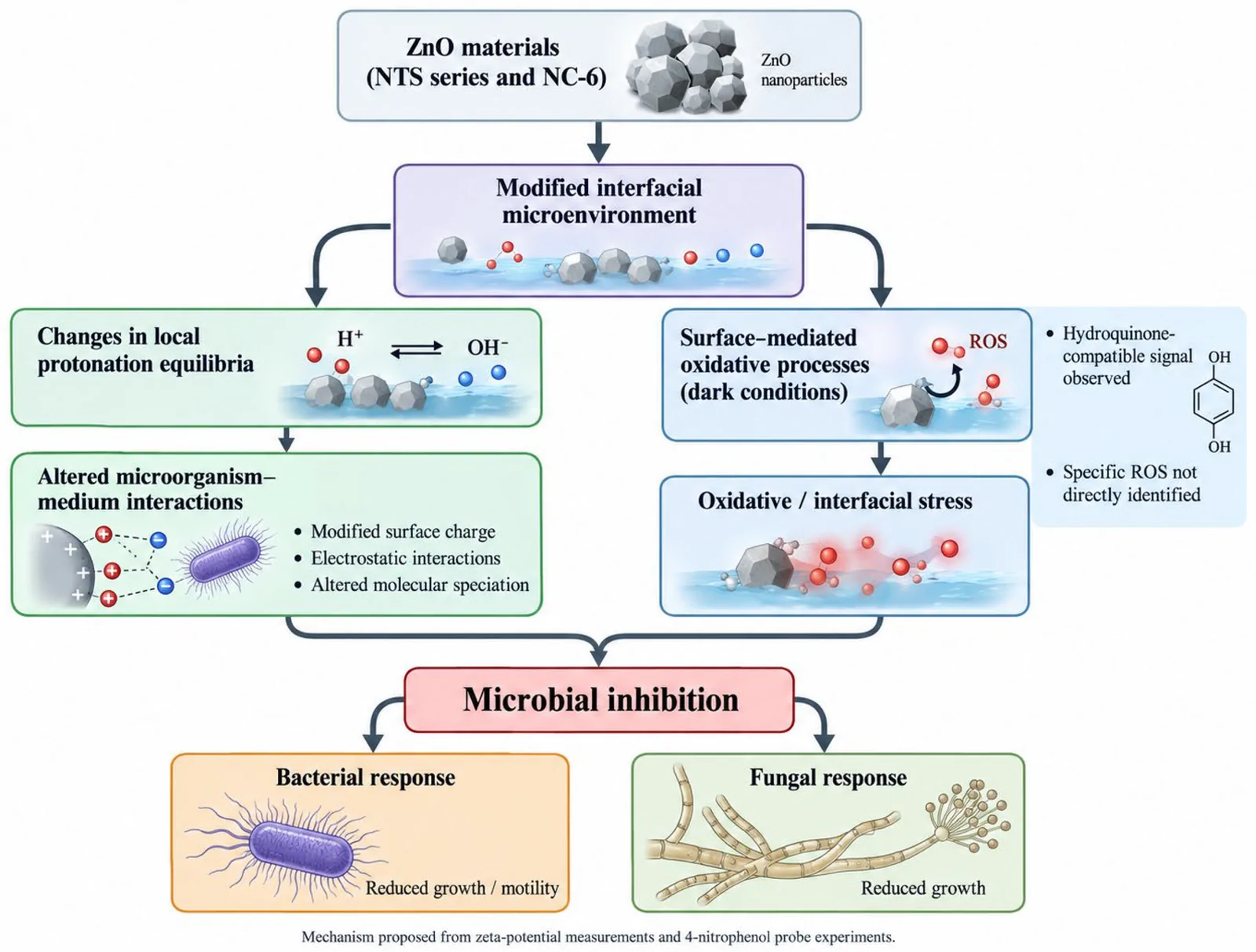
Proposed surface-mediated mechanism of microbial inhibition by ZnO materials.

Based on the obtained results, a surface-mediated mechanism is proposed to explain the inhibitory activity of the ZnO materials against the evaluated microorganisms, see (Figure 9). The experiments performed with 4-nitrophenol show that, even in the absence of irradiation, contact with the samples modifies the chemical environment at the solid-solution interface, favoring changes in the protonation equilibrium of 4-NP. This behavior demonstrates that the NTS surfaces do not act as inert phases but can locally modify the speciation of molecules present in the surrounding medium.

Changes in these protonation equilibria may have biological implications because the protonation state of functional groups present in biomolecules and cellular structures influences their charge, conformation, and electrostatic interactions. Therefore, surface-induced changes in the ZnO interfacial microenvironment could alter microorganism–medium interactions and contribute to affecting processes related to growth, proliferation, or motility. This first pathway is proposed as a potential consequence of the ability of the material to modify the local chemical environment rather than as a directly demonstrated cellular effect.

In parallel, the spectral modifications observed in the 280–290 nm region, which are compatible with the formation of hydroxylated aromatic species such as hydroquinone, suggest that surface-mediated oxidative transformations may also occur under dark conditions. The formation of this type of species is consistent with oxidative pathways reported for 4-NP and provides indirect evidence that the ZnO surface may promote oxidative processes even in the absence of irradiation. Since these conditions were selected to approximate those used during the microbial inhibition assays, such processes are proposed as a second potential pathway of action.

Under this pathway, oxidative species generated or promoted at the interface could induce oxidative and/or interfacial stress in microbial cells and, consequently, alter their interaction with the material and affect processes associated with growth, proliferation, or motility. However, because the specific oxidizing species were not directly identified in this study, this stage should be considered a plausible mechanistic interpretation rather than the definitive identification of a particular ROS.

Overall, the proposed mechanism considers two potentially complementary processes: (i) modification of the interfacial microenvironment, capable of altering protonation equilibria and microorganism–medium interactions, and (ii) surface-mediated oxidative processes that could induce interfacial or oxidative stress and contribute to microbial inhibition. The relative contribution of each pathway would depend on both the surface characteristics of each material and the response of the microorganism evaluated.

Table 5 compares the present study with recent reports on plant-assisted synthesis of ZnO nanoparticles. Previous studies have mainly focused on conventional antimicrobial activity, antioxidant performance, or photocatalytic degradation, with particle morphologies ranging from quasi-spherical to irregular or coarse structures [42,43,44,45]. In comparison, the present work evaluates a broader set of post-synthesis conditions and combines structural, morphological, textural, optical, and electrokinetic characterization with antimicrobial and swarming-motility assays. A distinctive feature of this study is the evaluation of the relationship between the physicochemical evolution of the ZnO materials and their biological response, particularly the marked anti-swarming activity observed for **NTS-4**. In addition, the use of zeta-potential measurements and 4-NP as a molecular probe provides complementary information on the solid-solution interfacial behavior, allowing the biological activity to be discussed in terms of a proposed surface-mediated mechanism rather than solely on the basis of particle size or morphology.

## 5. Conclusions

In this study, ZnO nanoparticles were successfully obtained through a bio-assisted synthesis using a hydroalcoholic extract of *T. spathacea*. The characterization results confirmed the formation of the hexagonal wurtzite-like ZnO phase in all biosynthesized materials, while also demonstrating that the thermal treatment modified the surface-associated organic functionalities, phase composition, morphology, aggregation state, and textural properties. In contrast, the crystallite size remained relatively constant among the biosynthesized samples, indicating that the differences observed in biological performance were not primarily associated with crystallite size.

The antimicrobial evaluation showed that all biosynthesized ZnO nanoparticles exhibited growth-inhibitory activity against the microorganisms evaluated, with *P. mirabilis* 884 showing the greatest susceptibility, with a MIC of 320 μg/mL. The subsequent swarming assay demonstrated that the nanoparticles also affected bacterial motility, and the magnitude of this effect depended on the synthesis conditions. **NTS-4**, obtained at 200 °C, showed the highest anti-swarming activity at the lowest concentration tested, reaching 96.69% inhibition after 24 h and 97.58% after 48 h.

Overall, the results indicate that the biological performance of the bio-assisted ZnO nanoparticles cannot be explained by crystallite size or surface area alone. Instead, the observed anti-swarming response was associated with differences in the ZnO crystalline phase, surface-associated organic species, morphology, aggregation, and textural characteristics. However, the present results do not allow the individual contribution of these physicochemical features to the biological response to be established. The results further suggest that intermediate thermal treatment may provide a favorable balance between ZnO formation and retention of plant-derived surface components.

These findings support the potential of *T. spathacea*-mediated ZnO nanoparticles as materials with antibacterial and anti-swarming properties. However, further studies are required to clarify the molecular mechanisms underlying these effects, quantitatively characterize particle morphology and aggregation, evaluate cytotoxicity, and determine their performance in more complex biological models before considering potential biomedical or antimicrobial applications.

To conclude, the results obtained suggest that ZnO nanoparticles synthesized by plant extracts could be explored in antimicrobial coatings and surfaces, as reported in previous studies. However, it is necessary to evaluate their stability, biocompatibility and efficacy under practical conditions. Likewise, advances in integrated intelligent sensing systems, such as sensors based on WO_3_/CNTs and MWCNTs–InSe, offer prospects for the development of functional materials. Although the present study does not evaluate detection applications, future research could explore the integration of these biosynthesized nanoparticles into microbial monitoring platforms, after validation of their sensitivity, selectivity and reproducibility [46,47,48].

## Figures and Tables

**Table 1:** Experimental conditions for the bioassisted synthesis of ZnO nanoparticles using hydroalcoholic extract of *T. spathacea* (NTS). ZnO product of combustion (NC).

Experiment Number	Drying Temperature (°C)	Drying Time (h)	Nomenclature
1	Room temperature	18	**NTS-1**
2	50	18	**NTS-2**
3	100	18	**NTS-3**
4	200	4	**NTS-4**
5	500	4	**NTS-5**
6	500	4	**NC-6**

**Table 2:** Surface area, pore volume, pore size, crystallite size, lattice parameters and zeta potential of ZnO nanoparticles.

Experiment	Surface Area (m^2^/g)	Pore Volume (cm^3^/g)	Pore Size (nm)	Crystal Size (nm)	a (Å)	c (Å)	V (Å3)	Zeta Potential (mV)
**NTS-1**	34.2	0.38	44.6	15.7	3.2572	5.2287	48.0415	−3.09 ± 0.58
**NTS-2**	30.1	0.34	42.6	15.6	3.2572	5.1876	47.6642	−13.03 ± 0.64
**NTS-3**	32.6	0.34	42.4	15.6	3.2502	5.2198	47.7545	−9.43 ± 0.45
**NTS-4**	28.6	0.38	53.3	15.6	3.2572	5.1891	47.6776	−8.54 ± 0.14
**NTS-5**	11.3	0.24	85.9	15.5	3.2295	5.1891	46.8682	−3.90 ± 0.19
**NC-6**	0.63	0.0008	5.3	70.3	3.2572	5.2154	47.9195	−4.12 ± 0.13

**Table 4:** Percentage of inhibition of nanoparticles at 24 and 48 h of incubation.

Time (h)		24 h		48 h	
		**Zone Mobility** **(mm)**	**% Inhibition**	**Zone Mobility** **(mm)**	**% Inhibition**
**C (+)**		**30.3**		**45.64**	
**Sample**	**Concentration** **(mg/mL)**				
**NTS-1**	3.2	13.2	56.43	39	14.54
**NTS-1**	1.6	21	30.69	43.6	4.46
**NTS-1**	0.8	24.4	19.47	48.2	0
**NTS-1**	0.4	24.9	17.82	49	0
**NTS-2**	3.2	NP	100	NP	100
**NTS-2**	1.6	NP	100	NP	100
**NTS-2**	0.8	NP	100	NP	100
**NTS-2**	0.4	26.2	30.03	39.3	13.89
**NTS-3**	3.2	NP	100	NP	100
**NTS-3**	1.6	NP	100	NP	100
**NTS-3**	0.8	NP	100	NP	100
**NTS-3**	0.4	8.36	72.41	13.8	69.76
**NTS-4**	3.2	NP	100	NP	100
**NTS-4**	1.6	NP	100	NP	100
**NTS-4**	0.8	NP	100	NP	100
**NTS-4**	0.4	1	96.69	1.1	97.58
**NTS-5**	3.2	NP	100	NP	100
**NTS-5**	1.6	NP	100	NP	100
**NTS-5**	0.8	NP	100	NP	100
**NTS-5**	0.4	11.1	63.36	21.26	53.42
**NC-6**	3.2	NP	100	NP	100
**NC-6**	1.6	NP	100	NP	100
**NC-6**	0.8	5.4	82.17	7	84.23
**NC-6**	0.4	21	30.69	38	16.73

ZnO NPs obtained by bioassisted synthesis using hydroalcoholic extract of *T. spathacea*, where **NTS-1**: room temperature; **NTS-2**: 50 °C; **NTS-3**: 100 °C; **NTS-4**: 200 °C; **NTS-5**: 500 °C; **NC-6**: Combustion at 500 °C. **C+**: positive control (Amikacin at 0.37 μg/mL). **NP**: without mobility.

**Table 5:** Comparison of the properties and applications of ZnO NPs.

Zn Source	Plant Source	Particle Size	Morphology	Application	Drying Temperature (°C)	Reference
Zinc acetate dihydrate	*Pluchea indica* leaves	21.9 nm (average, XRD)	Spherical, widely dispersed	Antimicrobial activity Photocatalytic degradation.	80	[42]
Zinc acetate dihydrate	*Viscum album* leaves (methanol extract)	13.5 nm (average, TEM)	Quasi-spherical	Antibacterial and antioxidant activity.		[43]
Zinc acetate dihydrate	*Punica granatum* skin and spent coffee grounds	115.17 nm (average, XRD)	Coarse particle formation of non-homogenized nanoparticles	Antibacterial activity and biocompatibility.		[44]
Zinc acetate	*Delonix elata* extract	30.1 nm (average Crystallite Size, XRD)	Predominantly spherical	Antibacterial activity.	400	[45]
Zinc acetate dihydrate	*Tradescantia spathacea* leaves (hydroalcoholic extract)	Crystallite size 15.61 (average, XRD)	Triangular sheets and stacked flake-like structures	Antibacterial activity, motility inhibition in swarming; degradation of 4-Nitrophenol.	25, 50, 100, 200 and 500 °C	Current work

## Data Availability

The data presented in this research are available on request from the corresponding author.

## Supplementary Materials

The following supporting information can be downloaded at https://www.mdpi.com/article/10.3390/nano16211366/s1, Figure S1, Evaluation of swarming-type antivirulence activity in *P. mirabilis* 884 by ZnO NPs obtained through bio-assisted synthesis with a hydroalcoholic extract of *T. spathacea* (NTS) over 24 h at concentrations of 0.4, 0.8, 1.6, and 3.2 mg/mL. NTS-1: room temperature; NTS-2: 50 °C; NTS-3: 100 °C; NTS-4: 200 °C; NTS-5: 500 °C; NC-6: Combustion at 500 °C; and C+: Amikacin; Figure S2, Evaluation of swarming-type antivirulence activity in *P. mirabilis* 884 by ZnO NPs obtained through bio-assisted synthesis with a hydroalcoholic extract of *T. spathacea* (NTS) over 48 h at concentrations of 0.4, 0.8, 1.6, and 3.2 mg/mL. NTS-1: room temperature; NTS-2: 50 °C; NTS-3: 100 °C; NTS-4: 200 °C; NTS-5: 500 °C; NC-6: Combustion at 500 °C; and C+: Amikacin.

## References

[1] Cui Y., Wei Q., Park H., Lieber C.M. (2001). Nanowire nanosensors for highly sensitive and selective detection of biological and chemical species. Science.

[2] Zarzycki A. (2014). At Source of Nanotechnology. TecnoLógicas.

[3] Patra J.K., Gouda S. (2013). Application of Nanotechnology in Textile Engineering: An Overview. J. Eng. Technol. Res..

[4] Dastjerdi R., Montazer M. (2010). A Review on the Application of Inorganic Nano-Structured Materials in the Modification of Textiles: Focus on Anti-Microbial Properties. Colloids Surf. B Biointerfaces.

[5] Saha R., Munshi M.H., Akter M., Shikder A.A.R., Islam T. (2025). Synthesis Techniques, Fundamental Properties, and Emerging Applications of Nanocomposites: A Comprehensive Review. SPE Polym..

[6] Thostenson E.T., Ren Z., Chou T.-W. (2001). Advances in the Science and Technology of Carbon Nanotubes and Their Composites: A Review. Compos. Sci. Technol..

[7] Azizi S., Mohamad R., Shahri M.M. (2017). Green Microwave-Assisted Combustion Synthesis of Zinc Oxide Nanoparticles with *Citrullus colocynthis* (L.) Schrad: Characterization and Biomedical Applications. Molecules.

[8] Azam A., Ahmed A.S., Oves M., Khan M.S., Habib S.S., Memic A. (2012). Antimicrobial Activity of Metal Oxide Nanoparticles against Gram-Positive and Gram-Negative Bacteria: A Comparative Study. Int. J. Nanomed..

[9] Stoimenov P.K., Klinger R.L., Marchin G.L., Klabunde K.J. (2002). Metal Oxide Nanoparticles as Bactericidal Agents. Langmuir.

[10] Emami-Karvani Z. (2012). Antibacterial Activity of ZnO Nanoparticle on Gram-Positive and Gram-Negative Bacteria. Afr. J. Microbiol. Res..

[11] Raghupathi K.R., Koodali R.T., Manna A.C. (2011). Size-Dependent Bacterial Growth Inhibition and Mechanism of Antibacterial Activity of Zinc Oxide Nanoparticles. Langmuir.

[12] Sangeetha G., Rajeshwari S., Venckatesh R. (2011). Green Synthesis of Zinc Oxide Nanoparticles by Aloe barbadensis Miller Leaf Extract: Structure and Optical Properties. Mater. Res. Bull..

[13] Janaki A.C., Sailatha E., Gunasekaran S. (2015). Synthesis, Characteristics and Antimicrobial Activity of ZnO Nanoparticles. Spectrochim. Acta Part A.

[14] Iravani S. (2011). Green Synthesis of Metal Nanoparticles Using Plants. Green Chem..

[15] Mittal A.K., Chisti Y., Banerjee U.C. (2013). Synthesis of Metallic Nanoparticles Using Plant Extracts. Biotechnol. Adv..

[16] Lansdown A.B.G. (1996). Zinc in the Healing Wound. Lancet.

[17] Lin P.-H., Sermersheim M., Li H., Lee P.H.U., Steinberg S.M., Ma J. (2017). Zinc in Wound Healing Modulation. Nutrients.

[18] Smijs T.G., Pavel S. (2011). Titanium Dioxide and Zinc Oxide Nanoparticles in Sunscreens: Focus on Their Safety and Effectiveness. Nanotechnol. Sci. Appl..

[19] Nohynek G.J., Lademann J., Ribaud C., Roberts M.S. (2007). Grey Goo on the Skin? Nanotechnology, Cosmetics, and Sunscreen Safety. Crit. Rev. Toxicol..

[20] García-Fernández A., Pérez-González M.Z., Jiménez-Arellanes M.A. (2026). A Systematic Review of *Tradescantia spathacea* (Purple Maguey): From Traditional Use to Pharmacological Research. Rev. Chapingo Ser. Hortic..

[21] Tan J.B.L., Lim Y.Y., Lee S.M. (2015). Antioxidant and Antibacterial Activity of *Rhoeo spathacea* (Swartz) Stearn Leaves. J. Food Sci. Technol..

[22] Rosales-Reyes T., De La Garza M., Arias-Castro C., Rodríguez-Mendiola M., Fattel-Fazenda S., Arce-Popoca E., Hernández-García S., Villa-Treviño S. (2007). Aqueous Crude Extract of Rhoeo discolor, a Mexican Medicinal Plant, Decreases the Formation of Liver Preneoplastic Foci in Rats. J. Ethnopharmacol..

[23] Sriwanthana B., Treesangsri W., Boriboontrakul B., Niumsakul S., Chavalittumrong P. (2007). In Vitro Effects of Thai Medicinal Plants on Human Lymphocyte Activity. Songklanakarin J. Sci. Technol..

[24] Xu J., Huang Y., Zhu S., Abbes N., Jing X., Zhang L. (2021). A Review of the Green Synthesis of ZnO Nanoparticles Using Plant Extracts and Their Prospects for Application in Antibacterial Textiles. J. Eng. Fibers Fabr..

[25] Mendes C.R., Dilarri G., Forsan C.F., Sapata V.M.R., Lopes P.R.M., de Moraes P.B., Montagnolli R.N., Ferreira H., Bidoia E.D. (2022). Antibacterial Action and Target Mechanisms of Zinc Oxide Nanoparticles against Bacterial Pathogens. Sci. Rep..

[26] Lithi I.J., Nakib K.I.A., Chowdhury A.M.S., Hossain M.S. (2025). A Review on the Green Synthesis of Metal (Ag, Cu, and Au) and Metal Oxide (ZnO, MgO, Co_3_O_4_, and TiO_2_) Nanoparticles Using Plant Extracts for Developing Antimicrobial Properties. Nanoscale Adv..

[27] Tan S.-P., Keng X.-Y., Lim C.-W.B., Tan H.-Y. (2024). Traditional Uses, Phytochemistry and Pharmacological Activities of *Tradescantia spathacea*. Rec. Nat. Prod..

[28] López A.R.A., De la Rosa L.E.S., Mosqueda S.L.C., Cortez J.C., Pavon A.A.S., Uribe A.C., Gómez C.M. (2025). Photocatalytic Properties of ZnO Synthesized by Solid-State Combustion: Structural Analysis and Degradation of 4-Nitrophenol. J. Basic Sci..

[29] Monshi A., Foroughi M.R., Monshi M.R. (2012). Modified Scherrer Equation to Estimate More Accurately Nano-Crystallite Size Using XRD. World J. Nano Sci. Eng..

[30] Gómez-Rivera A., Barredo-Hernández C.O., Santos-Vázquez S., Lobato-García C.E., Gallegos-García A.J., López-Rodríguez R., Alvarez L., Pérez-García M.D., González-Cortazar M., Torres-López J.L. (2026). Thorough Characterization of Two Sessein Derivatives with Potential Biological Activity. Molecules.

[31] Salazar-Piña D.T., Castro-Alarcón N., Moreno-Godínez M.E., Del Pilar Nicasio-Torres M., Pérez-Hernández J., Álvarez-Fitz P. (2017). Antibacterial and Anti-inflammatory Activity of Extracts and Fractions from Agave cupreata. Int. J. Pharmacol..

[32] Araujo A.V., Quiroz M.A., Sánchez B.E., Martínez-Santos V., Ramírez M., Salazar R., Godínez M.E.M., Coronel Y.C., Maruri D.T., Cortazar M.G. (2026). Antibacterial and Antivirulence Activity of Lyophilized *Plumbago auriculata* Flower Extract against *Escherichia coli* and *Proteus mirabilis*. AppliedChem.

[33] Veisi H., Karmakar B., Tamoradi T., Tayebee R., Sajjadifar S., Lotfi S., Maleki B., Hemmati S. (2021). Bio-Inspired Synthesis of Palladium Nanoparticles Fabricated Magnetic Fe_3_O_4_ Nanocomposite over Fritillaria imperialis Flower Extract as an Efficient Recyclable Catalyst for the Reduction of Nitroarenes. Sci. Rep..

[34] Jan H., Shah M., Usman H., Khan M.A., Zia M., Hano C., Abbasi B.H. (2020). Biogenic Synthesis and Characterization of Antimicrobial and Antiparasitic Zinc Oxide (ZnO) Nanoparticles Using Aqueous Extracts of the Himalayan Columbine (*Aquilegia pubiflora*). Front. Mater..

[35] Karam S.T., Abdulrahman A.F. (2022). Green Synthesis and Characterization of ZnO Nanoparticles by Using Thyme Plant Leaf Extract. Photonics.

[36] Selim Y.A., Azb M.A., Ragab I., El-Azim M.H.M.A. (2020). Green Synthesis of Zinc Oxide Nanoparticles Using Aqueous Extract of Deverra tortuosa and Their Cytotoxic Activities. Sci. Rep..

[37] Ehsani A., Ehsani I., Obut A. (2021). Preparation of Different Zinc Compounds from a Smithsonite Ore through Ammonia Leaching and Subsequent Heat Treatment. Physicochem. Probl. Miner. Process..

[38] Thommes M., Kaneko K., Neimark A.V., Olivier J.P., Rodriguez-Reinoso F., Rouquerol J., Sing K.S.W. (2015). Physisorption of Gases, with Special Reference to the Evaluation of Surface Area and Pore Size Distribution (IUPAC Technical Report). Pure Appl. Chem..

[39] Alejandro-López A.R., Serrano de la Rosa L.E., Castillejos-Mosqueda S.L., Córdova-Pérez G.E., Cerna-Cortez J.R., Gómez C.M., Silahua-Pavón A.A., Saavedra-Díaz R.O., Godavarthi S., Cervantes-Uribe A. (2025). Role of Surface Charge in the Speciation and Photocatalytic Degradation of 4-Nitrophenol Using ZnO–CeO_2_–WO_3_ Photocatalysts. J. Compos. Sci..

[40] Aman D., Abdel-Azim S., Said S., Mohamed S.G. (2022). Facile Synthesis of ZnMoO_4_/AlPO_4_-5 Nanorod Composites as Visible-Light-Driven Photocatalysts and High-Performance Energy Storage Materials. RSC Adv..

[41] Sirelkhatim A., Mahmud S., Seeni A., Kaus N.H.M., Ann L.C., Bakhori S.K.M., Hasan H., Mohamad D. (2015). Review on zinc oxide nanoparticles: Antibacterial activity and mechanism of toxicity. Nano-Micro Lett..

[42] Al-Askar A.A., Hashem A.H., Elhussieny N.I., Saied E. (2023). Green Biosynthesis of Zinc Oxide Nanoparticles Using Pluchea indica Leaf Extract: Antimicrobial and Photocatalytic Activities. Molecules.

[43] Mushtaq W., Ishtiaq M., Maqbool M., Mazhar M.W., Casini R., Abd-ElGawad A.M., Elansary H.O. (2023). Green Synthesis of Zinc Oxide Nanoparticles Using Viscum album Extracts: Unveiling Bioactive Compounds, Antibacterial Potential, and Antioxidant Activities. Plants.

[44] Abdelmigid H.M., Hussien N.A., Alyamani A.A., Morsi M.M., AlSufyani N.M., Kadi H.A. (2022). Green Synthesis of Zinc Oxide Nanoparticles Using Pomegranate Fruit Peel and Solid Coffee Grounds vs. Chemical Method of Synthesis, with Their Biocompatibility and Antibacterial Properties Investigation. Molecules.

[45] Nanthakumar J., Palanisamy Y., Palanisamy S., Karuppusamy M., Raja R., Abbas M., Alagarsamy A., Rahman M.Z. (2025). Eco-Friendly Synthesis of ZnO Nanoparticles Using Delonix elata Extract with Enhanced Antibacterial Activity. RSC Adv..

[46] Rohani R., Dzulkharnien N.S.F., Harun N.H., Ilias I.A. (2022). Green Approaches, Potentials, and Applications of Zinc Oxide Nanoparticles in Surface Coatings and Films. Bioinorg. Chem. Appl..

[47] Li Y., Liao C., Tjong S.C. (2020). Recent Advances in Zinc Oxide Nanostructures with Antimicrobial Activities. Int. J. Mol. Sci..

[48] Gao M., Zhang W., Guo J., Fu L. (2026). Integrated Intelligent Sensing System with WO_3_/CNTs Sensor for on-Site Gas Detection. Sens. Actuators B Chem..

