# Supplementary material for: Thermal Processing as a Key Factor in the Surface Chemistry and Preliminary Antimicrobial Activity of ZnO Nanoparticles Synthesized Using Tradescantia spathacea Sw., Extract
Source: Nanomaterials (Basel). 2026 Oct 26;16(21):1366. doi: 10.3390/nano16211366 (PMC13765103; doi:10.3390/nano16211366)
Supplement: Supplementary file 1 [file nanomaterials-16-01366-s001.zip › nanomaterials-4618916-supplementary.pdf]

Thermal Processing as a Key Factor in the Surface Chemistry and Preliminary Antimicrobial Activity of ZnO Nanoparticles Synthesized Using *Tradescantia spathacea* Sw. Extract.

Figure S1

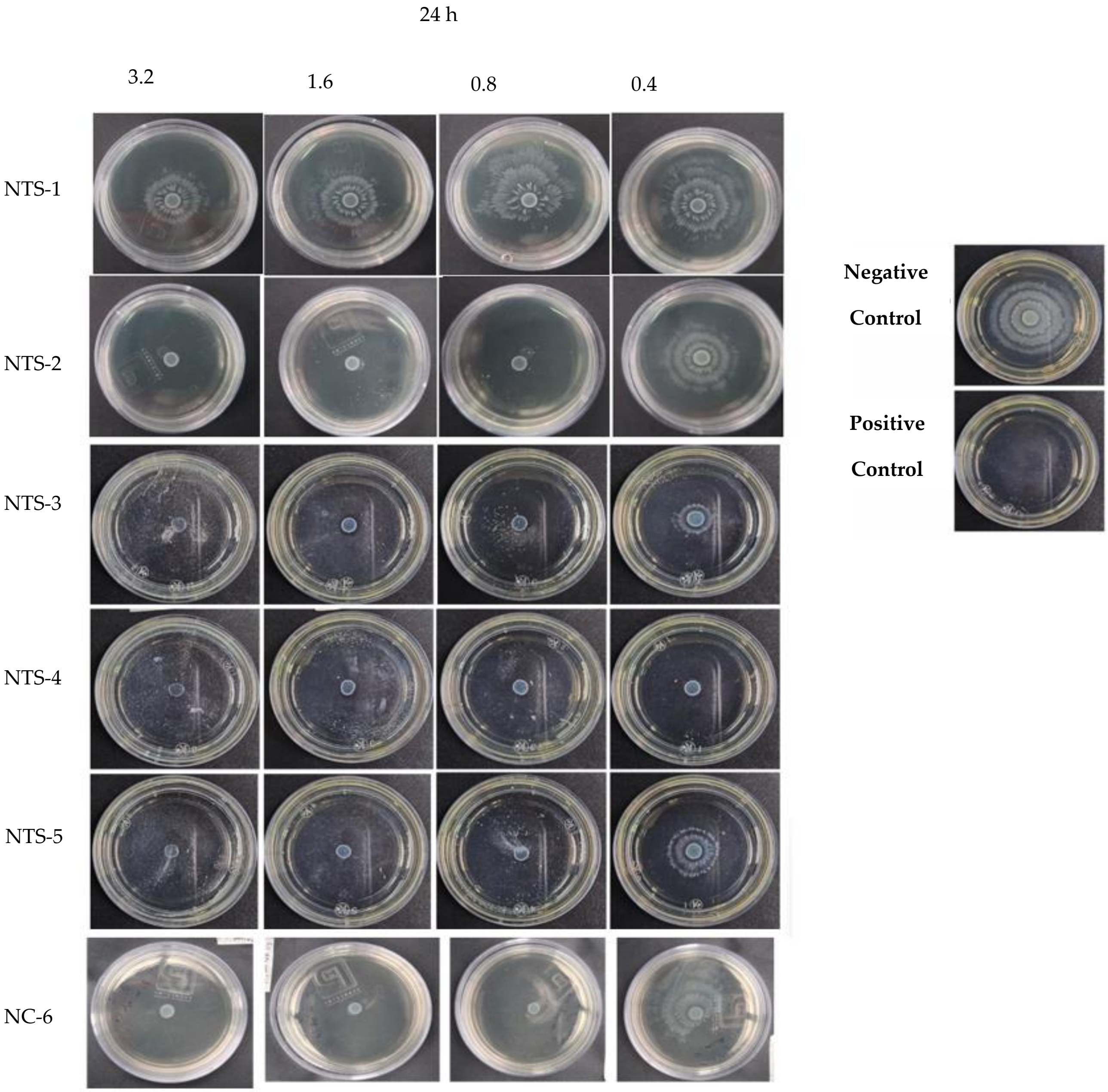

Figure S1. Evaluation of swarming-type antivirulence activity in *P. mirabilis* 884 by ZnO NPs obtained through bio-assisted synthesis with a hydroalcoholic extract of *T. spathacea* (NTS) over 24 hours at concentrations of 0.4, 0.8, 1.6, and 3.2 mg/mL. **NTS-1**: room temperature; **NTS-2**: 50 °C; **NTS-3**: 100 °C; **NTS-4**: 200 °C; **NTS-5**: 500 °C; **NC-6**: Combustion at 500 °C; and **C+**: Amikacin

Figure S2

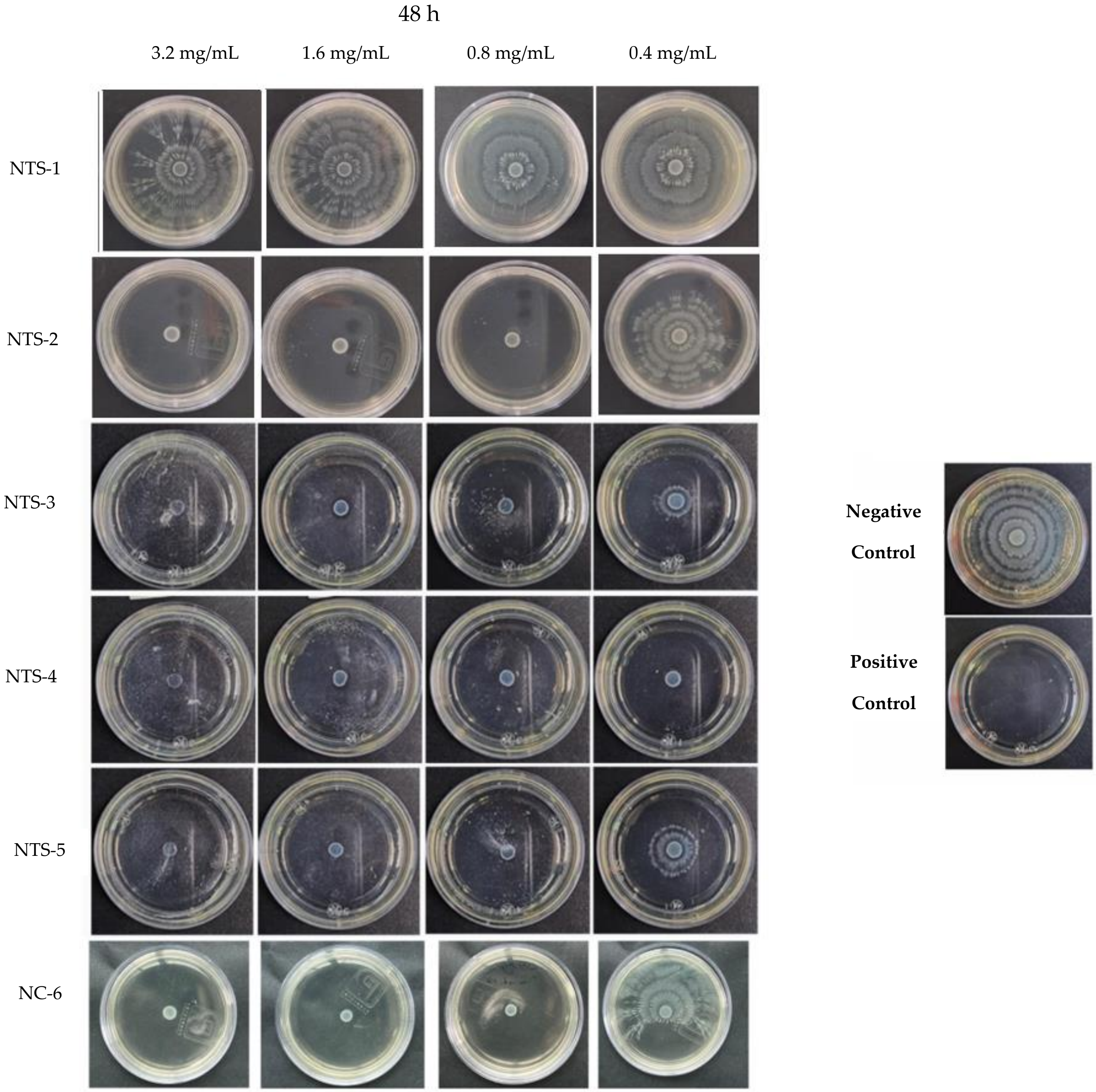

Figure S2. Evaluation of swarming-type antivirulence activity in *P. mirabilis* 884 by ZnO NPs obtained through bio-assisted synthesis with a hydroalcoholic extract of *T. spathacea* (NTS) over 48 hours at concentrations of 0.4, 0.8, 1.6, and 3.2 mg/mL. **NTS-1**: room temperature; **NTS-2**: 50 °C; **NTS-3**: 100 °C; **NTS-4**: 200 °C; **NTS-5**: 500 °C; **NC-6**: Combustion at 500 °C; and **C+**: Amikacin
